# Methyl Viologens of Bis‐(4’‐Pyridylethynyl)Arenes – Structures, Photophysical and Electrochemical Studies, and their Potential Application in Biology

**DOI:** 10.1002/chem.202200753

**Published:** 2022-05-30

**Authors:** Goutam Kumar Kole, Marta Košćak, Anissa Amar, Dragomira Majhen, Ksenija Božinović, Zlatko Brkljaca, Matthias Ferger, Evripidis Michail, Sabine Lorenzen, Alexandra Friedrich, Ivo Krummenacher, Michael Moos, Holger Braunschweig, Abdou Boucekkine, Christoph Lambert, Jean‐François Halet, Ivo Piantanida, Klaus Müller‐Buschbaum, Todd B. Marder

**Affiliations:** ^1^ Institut für Anorganische Chemie, and Institute for Sustainable Chemistry & Catalysis with Boron Julius-Maximilians-Universität Würzburg Am Hubland 97074 Würzburg Germany; ^2^ Department of Chemistry College of Engineering and Technology SRM Institute of Science and Technology, SRM Nagar Kattankulathur Tamil Nadu 603203 India; ^3^ Ruđer Bošković Institute 10000 Zagreb Croatia; ^4^ Laboratoire de Physique et Chimie Quantiques Université Mouloud Mammeri Tizi Ouzou 15000 Tizi-Ouzou Algeria; ^5^ Institut für Organische Chemie Julius-Maximilians-Universität Würzburg Am Hubland 97074 Würzburg Germany; ^6^ Univ Rennes, Ecole Nationale Supérieure de Chimie de Rennes, CNRS, Institut des Sciences Chimiques de Rennes UMR 6226 35000 Rennes France; ^7^ CNRS-Saint-Gobain-NIMS IRL 3629 Laboratory for Innovative Key Materials and Structures (LINK) National Institute for Materials Science (NIMS) Tsukuba 305-0044 Japan; ^8^ Institut für Anorganische und Analytische Chemie Justus-Liebig-Universität Gießen Heinrich-Buff-Ring 17 35392 Gießen Germany

**Keywords:** cell imaging, DNA/RNA binding, methyl viologen, singlet oxygen, two-photon absorption

## Abstract

A series of bis‐(4’‐pyridylethynyl)arenes (arene=benzene, tetrafluorobenzene, and anthracene) were synthesized and their bis‐*N*‐methylpyridinium compounds were investigated as a class of π‐extended methyl viologens. Their structures were determined by single crystal X‐ray diffraction, and their photophysical and electrochemical properties (cyclic voltammetry), as well as their interactions with DNA/RNA were investigated. The dications showed bathochromic shifts in emission compared to the neutral compounds. The neutral compounds showed very small Stokes shifts, which are a little larger for the dications. All of the compounds showed very short fluorescence lifetimes (<4 ns). The neutral compound with an anthracene core has a quantum yield of almost unity. With stronger acceptors, the analogous bis‐*N*‐methylpyridinium compound showed a larger two‐photon absorption cross‐section than its neutral precursor. All of the dicationic compounds interact with DNA/RNA; while the compounds with benzene and tetrafluorobenzene cores bind in the grooves, the one with an anthracene core intercalates as a consequence of its large, condensed aromatic linker moiety, and it aggregates within the polynucleotide when in excess over DNA/RNA. Moreover, all cationic compounds showed highly specific CD spectra upon binding to ds‐DNA/RNA, attributed to the rare case of forcing the planar, achiral molecule into a chiral rotamer, and negligible toxicity toward human cell lines at ≤10 μM concentrations. The anthracene‐analogue exhibited intracellular accumulation within lysosomes, preventing its interaction with cellular DNA/RNA. However, cytotoxicity was evident at 1 μM concentration upon exposure to light, due to singlet oxygen generation within cells. These multi‐faceted features, in combination with its two‐photon absorption properties, suggest it to be a promising lead compound for development of novel light‐activated theranostic agents.

## Graphical abstract

**Methyl‐viologens containing** π‐extended bis(*N*‐methylpyridiniumethynyl)arenes (arene=benzene, tetrafluorobenzene, anthracene) were crystallographically characterized and their electrochemical properties and one‐ and two‐photon absorption and fluorescence spectra were measured. With DNA/RNA, while the benzene and tetrafluorobenzene analogues bind in grooves, the anthracene analogue intercalates due to its large aromatic moiety, and aggregates within the polynucleotide when in excess over DNA/RNA. The latter compound localizes within lysosomes, preventing its interaction with cellular DNA/RNA, but exhibited cytotoxicity upon photoexcitation, due to ^1^O_2_ generation within cells.

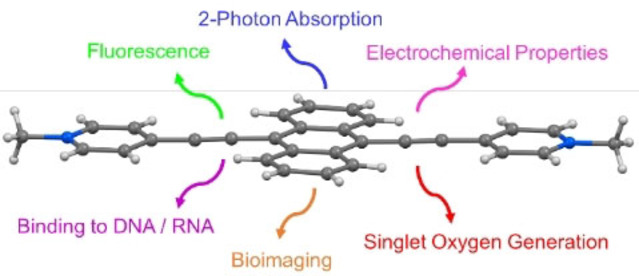

## Introduction

Viologens are N,N’‐di‐alkylated 4,4’‐bipyridinium salts, which have been extensively investigated in the past, and have attracted more attention recently for various reasons.[^1^, ^2^] Such compounds often possess three stable redox states, and act as efficient electron accepting capabilities (oxidizing agents). “Paraquat”, for example, N,N’‐dimethyl‐4,4’‐dipyridyl dichloride (MV^2+^), undergoes two reversible reductions to a radical‐cation (MV^C+^) and a neutral species (MV). Consequently, the colour changes from colourless (MV^2+^) to blue‐violet (MV^C+^), and finally to yellow‐brown (MV).^[1]^ Depending on the counter‐anions, and the substituents at the nitrogen atoms, the colour of the viologen compounds can change dramatically. Moreover, the tunability of these substituents make them an important class of compounds for designing new materials that have numerous applications in electrochromic[^2^, ^3^] and photochromic materials.^[4]^ Furthermore, such materials have been explored over the years for applications in molecular shuttles and switches or machines,^[5]^ and recently in organic “green batteries”.^[6]^ Significant progress has been made to fine tune the electronic and photophysical properties of viologens by introducing further functionalisation, such as additional bridging or incorporation of a hetero‐atom. For example, sulfide‐bridged,^[7]^ phosphole‐bridged,^[8]^ germanium‐bridged,^[9]^ thiophene‐based,^[10]^ and π‐extended viologen^[11]^ compounds have been reported. Recently, we reported phenyl‐pyridyl‐fused boroles, a different class of compounds for which a unique B−N coordination mode was observed that led to dual fluorescence in solution.^[12]^

Polyaromatic hydrocarbon (PAH)‐based viologen compounds with extended π‐conjugation have attracted recent attention due to their interesting photophysical properties with various potential applications.^[13]^ Konishi and co‐workers reported a pyrene‐derived extended π‐conjugated, acceptor‐π‐acceptor type viologen compound which exhibited an emission in the ‘tissue transparent window’ (650–1100 nm), and a two‐photon absorption band at ca. 1000 nm with a large cross‐section. It was shown to have good performance in 3D imaging of mitochondria in living cells.^[14]^ We have also recently reported the synthesis of mono‐ and bis‐substituted pyridyl‐pyrene compounds, and their photophysical properties.^[15]^ The 2‐substituted and 2,7‐disubstituted pyridyl‐pyrene compounds were further methylated, and a detailed study including their structures, photophysical, electrochemical properties, and their interaction with DNA was reported with in‐depth theoretical calculations. We found that the 2,7‐disubstituted *N*‐methylated compound, an acceptor‐π‐acceptor system, can act not only as an oxidizing agent, but also an effective bio‐active compound, signifying its utility as a multi‐functional material.^[16]^ It is worth mentioning that this compound underwent either to a two‐electron reduction or to two closely spaced consecutive one‐electron reductions, leading to the formation of the neutral species, without producing the intermediate radical cation.

We also have shown that upon methylation, the dihedral angle between the pyridinium ring and the central aromatic moiety approaches zero.^[16]^ Such a flat aromatic structure can facilitate its stacking with nucleobases and consequently intercalation into DNA/RNA.^[17]^ The possibility of excimer formation enables the exploration of advanced applications, such as the detection of DNA/RNA interactions both as a single label,^[18]^ as well as excimer‐forming pairs.^[19]^ Moreover, some interesting applications rely on their interactions with DNA or RNA grooves^[20]^ or in combination with their turn‐on and ‐off excimers.^[21]^ Recently, we reported several bis‐triaryl boron‐containing tetracationic chromophores with oligo‐thiophene‐based, and other π‐extended aromatic linkers that exhibited one‐ and two‐photon absorption and useful cell imaging properties.^[22]^ Rod‐like dumbbell structures derived from such tetracationic compounds by modification of the central π‐conjugated aromatic linkers with alkyne moieties (e. g., bis(phenylethynyl)arene, arene=benzene, anthracene, etc.) showed distinctly different properties in fluorimetric and Raman sensing of their interactions with RNAs/DNAs.^[23]^ It is worth noting that bioimaging techniques are essential tools in biomedical research and medical diagnostics and, among various possible methods, most rely on fluorescence‐based imaging.^[24]^

Singlet oxygen (^1^O_2_), a metastable excited state of molecular oxygen, is one of the most prominent reactive oxygen species (ROS) having a relatively long lifetime, which can persist for over an hour in the gas phase, and 10^−6^–10^−3^ seconds in solution.^[25]^ It thus finds applications in a wide range of fields, for example, in synthetic chemistry,^[26]^ materials science,[^27^, ^28^] and photodynamic therapy (PDT).[^29^, ^30^] Singlet oxygen is generated via the energy transfer‐based photoexcitation of ground‐state oxygen, which is known as photosensitization,^[31]^ or via chemical reaction, wherein oxygen can be trapped by the formation of, for example, endoperoxides.[^27^, ^30a^, ^32^] Several aromatic chromophores, such as naphthalene, 2‐pyridones, and anthracene derivatives, have been reported to react with singlet oxygen to form the corresponding endoperoxides (EPOs).[^27^, ^30a^, ^32^, ^33^] Recently, we reported *tetra*‐donor‐ or acceptor‐substituted *ortho*‐perylenes, the first examples of perylenes substituted only at the *ortho* positions with donors or acceptors. One such compound with four diarylamino donors, (DPA)_4_‐Per was shown to be an effective photosensitizer for singlet oxygen generation.^[34]^ Heavy transition metal complexes of, for example, Pt(II), Ru(II), Ir(III), etc., are known to photosensitize oxygen.[^31b^, ^35^] As methyl pyridinium derivatives can be water soluble, they can serve as oxygen photosensitizers in aqueous media. Therefore, viologen based compounds offer potential advantages for such applications.^[32c]^

Almost two decades ago, one of our groups reported a series of bis(pyridylethynyl)arene compounds, with fluorinated and non‐fluorinated moieties, including their synthesis, crystal structures, liquid crystal phase behaviour, and absorption and emission characteristics.^[36]^ Several compounds of this series were employed as linkers for coordination polymers and MOFs by many groups.^[37]^ We anticipated that such linear, dipyridyl compounds with extended π‐conjugated linkers were very good candidates for investigating multi‐functional behaviour ranging from photophysical to electrochemical properties and application in biology. In addition, investigation of the energy barrier of their rotamers,^[38]^ in combination with circular dichroism (CD) studies, can provide an in‐depth understanding of such materials. While our manuscript was in preparation, a detailed study on bis‐*N*‐methylated (*o*/*m*/*p*) bis‐(pyridiniumethynyl)anthracenes, reported by Linker et al.,^[39]^ revealed intriguing photophysical and photochemical properties, their strong interaction with ds‐DNAs, and photo‐induced DNA damage, based on singlet oxygen production. However, it remained unclear whether the large arene, anthracene, is essential for these properties, and what biological applications there might be of such fluorescent, potentially bioactive dyes. We conducted a very detailed investigation on the multi‐faceted utility of methyl viologen compounds derived from bis‐(4’‐pyridylethynyl)arenes (arenes=benzene, tetrafluorobenzene, and anthracene, the latter one only having been reported very recently with a sulphate counter‐anion^[39]^), their crystal and molecular structures, photophysical properties including two‐photon absorption, electrochemical properties, and their applications in biology as promising dyes for DNA‐targeting and theranostic purposes. Herein, we present these results.

## Results and Discussion

**Scheme 1:**
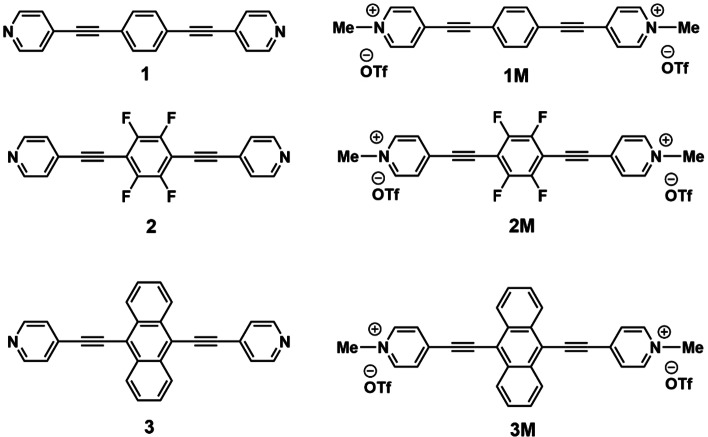
Chemical structures of the compounds discussed in this paper (**3M** was very recently reported with a sulphate counter‐anion,^[39]^ and herein, was synthesized and investigated with a triflate counter‐anion).

Using our neutral bis‐(4’‐pyridylethynyl)arenes, **1, 2** and **3**,^[36]^ we prepared their *N*‐methylated derivatives (π‐extended viologens) (Scheme 1) and carried out detailed studies of their photophysical, electrochemical and biological properties, especially binding to DNAs and RNAs.

## Syntheses

The dicationic compounds **1M**–**3M** were synthesized by reacting the respective neutral compounds^[36]^ with MeOTf in dry CH_2_Cl_2_ under an inert atmosphere, and single crystals were obtained by slow evaporation of concentrated solutions of the compounds in MeCN. The resulting dicationic compounds were characterized by single crystal X‐ray diffraction and various spectroscopic techniques. The compounds are stable, in general, and can be handled in air. Upon methylation, the ^1^H NMR signals for the protons of central phenylene or anthracene moieties, as well as for one of two types of pyridyl‐protons are somewhat down‐field shifted from those of the neutral compounds, as expected.^[16]^ For example, the signals for the pyridyl protons appear at 8.59 and 8.02 ppm for **1M** (Figure S3), and at 8.68 and 8.13 ppm for **2M** (Figure S7), and the signals for the central phenylene protons appear at 7.78 ppm for **1M**, which are down‐field shifted compared to the signals observed for **1** at 8.63 and 7.48 ppm (pyridyl protons), and 7.57 ppm (phenylene protons) (Figure S1), and 8.69 and 7.46 ppm (pyridyl protons) for **2** (Figures S5). It is noteworthy that the NMR spectra were recorded in CDCl_3_ for the neutral compounds (**1–3**) and in CD_3_CN for the dicationic compounds (**1M**–**3M**).

## Crystal and molecular structures of 1M, 2M, 3M and 3M’

**Figure 1:**
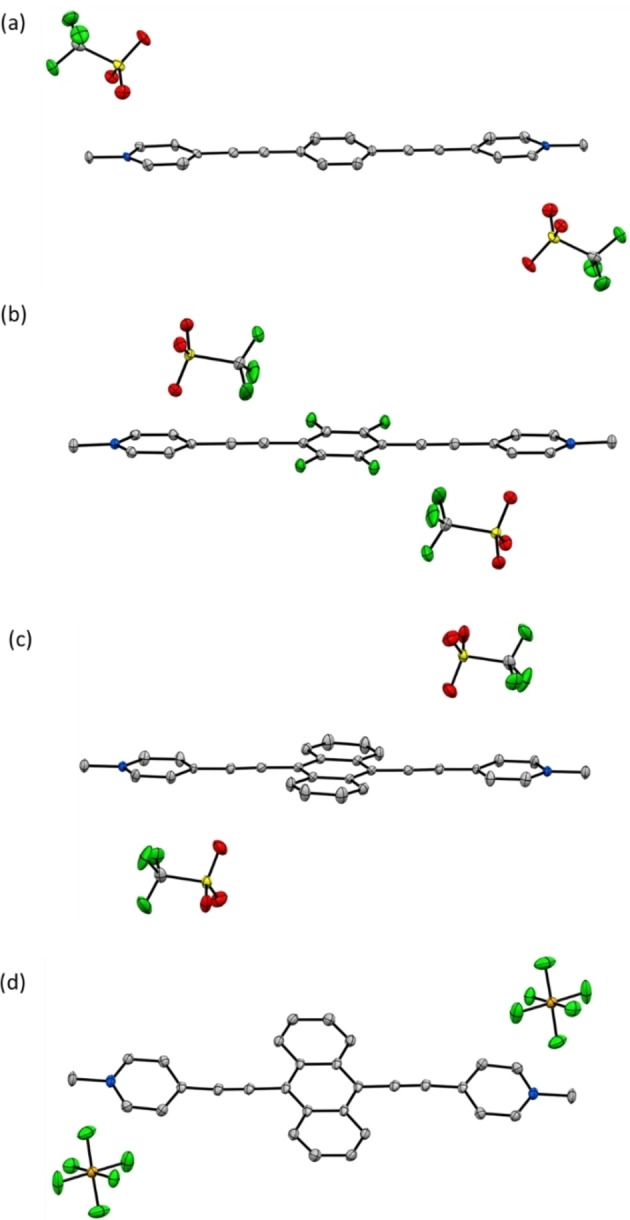
Molecular structures with displacement ellipsoids drawn at 50 % probability: a) **1M**; b) **2M**; c) **3M**; d) **3M’**. Colour code: grey – carbon, green – fluorine, yellow – sulphur, red – oxygen and blue – nitrogen.

Crystal structures of the neutral compounds (**1**–**3**) were reported previously.[^36^, ^37a^] While triflate anions are present in the dications **1M**, **2M** and **3M**, we also prepared the salt **3M’** with PF_6_^−^ anions. Crystallographic parameters of **1M**, **2M**, **3M** (space group *P*2_1_/*n*) and **3M’** (space group *P* $\bar{1}$ ) are listed in Table S1. All of the dications **1M**, **2M**, **3M** and **3M’** have inversion symmetry and, hence, the asymmetric unit of their crystal structures contains only half of the dication and one triflate (**1M**, **2M**, **3M**) or PF_6_^−^ (**3M’**) anion. The linear dications possess an almost planar conformation and show only small dihedral and torsion angles (2.0°–7.5°, Table S2) between the planes of the pyridinium rings and the central aromatic moieties (Figure 1). The torsion angles of **1M** (7.3°) and **2M** (5.1°) are in a similar range to those of their neutral analogues **1** (9° and 12.8°)^[37a]^ and **2** (2.5°).^[36]^ However, in the solid‐state structure of the neutral molecule **3**, the two pyridine rings have different orientations, rotated by 5.0° and 64.8° with respect to the anthracene plane,^[36]^ which is in stark contrast to the almost planar conformation of the dications **3M** (2.3°) and **3M’** (6.0°).

In the crystal structures of **1M**, **3M** and **3M’**, the packing of the dications is determined by π‐stacking interactions between the aromatic moieties. The dications of **1M** π‐stack in an offset parallel arrangement in which the cationic pyridinium groups interact with the central benzene moieties of the neighbouring molecules [interplanar separations (*d*) are *d*=3.354(3) and 3.446(2) Å] (Figures 1(a) and S16). The triflate anions interact with the dications via weak C−H⋅⋅⋅O, C−H⋅⋅⋅F and C⋅⋅⋅O interactions, and with other triflate anions via O⋅⋅⋅F interactions. A similar π‐stacking arrangement to that of **1M** is observed in **3M** and **3M’**, in which the dications form offset parallel stacks with the pyridinium moieties interacting with the central anthracene moieties of the neighbouring molecules on both sides (Figures 1c, d, S18 and S19). Interplanar separations are *d*=3.338(3) and 3.354(2) Å for **3M** and *d*=3.3685(15) and 3.4457(17) Å for **3M’**. Further C⋅⋅⋅C interactions are present between the terminal carbon atoms of the anthracene moieties of neighbouring stacks. In **3M**, triflate anions form weak intermolecular C−H⋅⋅⋅O, C−H⋅⋅⋅F, and C⋅⋅⋅F interactions with the dications and F⋅⋅⋅F interactions with other triflate anions. In **3M’**, PF_6_^−^ anions form C−H⋅⋅⋅F interactions and C⋅⋅⋅F contacts with the dications. In **2M**, all the dications are nearly coplanar and parallel, and are offset stacked via the pyridinium moieties of two neighbouring molecules [*d*=3.477(2) Å]. As the offset of 2.264(3) Å between neighbouring pyridinium moieties is large, this cannot be considered as a π‐stacking interaction. Two triflate anions are located in each interstice between stacks that are one above the other, forming weak C⋅⋅⋅F and C⋅⋅⋅O interactions with the tetrafluorobenzene moieties and the ethynyl groups of these stacks. In addition, triflate anions form weak C−H⋅⋅⋅O, C−H⋅⋅⋅F, and F⋅⋅⋅F interactions with the dications of side‐by‐side stacks (Figures 1b and S17).

## Photophysical properties

**Figure 2:**
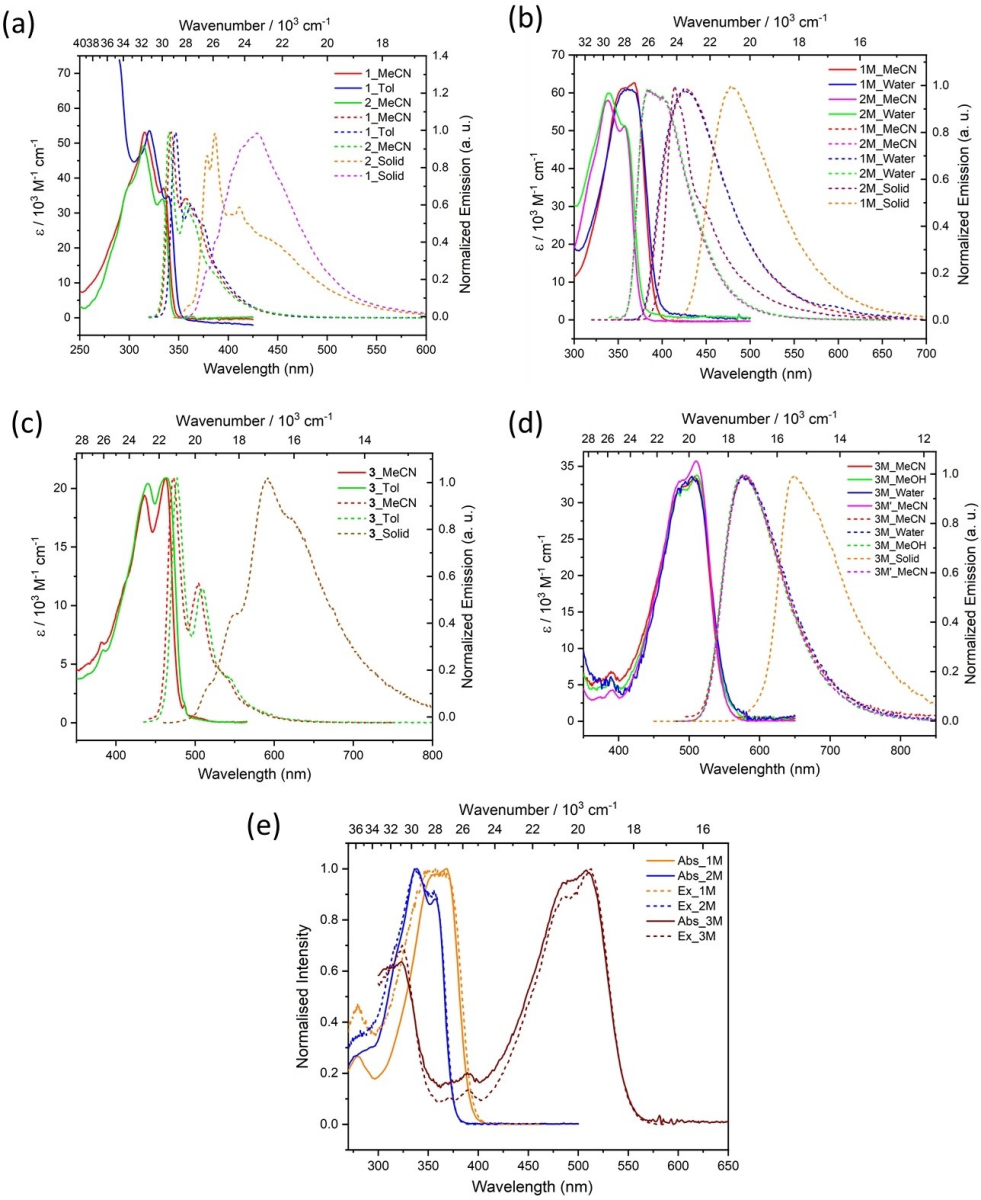
Absorption (solid lines) and emission (dashed lines) spectra of a) **1**, **2**; and b) **1M**, **2M** in MeCN (*λ*_ex_=*λ*_abs, max_); c) **3** (*λ*_ex_=435 nm) and d) **3M** (*λ*_ex_=485 nm) in MeCN, and other solvents, and in the solid state; e) absorption (solid lines) and excitation (dashed lines) spectra of **1M**, **2M**, and **3M** in MeCN showing excellent agreement.

Detailed photophysical properties of all compounds, in solution as well as in the solid state, are depicted in Figure 2. Absorption and emission spectra of the dicationic compounds (**1M**–**3M**) were measured in MeCN as well as in water. For **3M**, photophysical measurements were also carried out in MeOH. For the neutral compounds, absorption and emission spectra were measured in MeCN and toluene for **1** and **3**, and MeCN for **2**. Key photophysical data are summarised in Table 1. In order to provide a comparison of their absorption and emission characteristics, the data measured in MeCN are considered. The absorption and emission characteristics of **1** and **2** are very similar. They absorb at 335 nm (*ϵ*=37100 M^−1^ cm^−1^) and 333 nm (*ϵ*=34200 M^−1^ cm^−1^), respectively, and emit (*λ*_em_) at 342 nm and 340 nm, respectively. On the other hand, both the absorption of **3** at 464 nm (*ϵ*=20900 M^−1^ cm^−1^) and emission at 472 nm are comparatively red‐shifted. The absorption and emission spectra of all of the dications are red‐shifted compared to their neutral counterparts, as was observed earlier for the pyrene compounds.^[16]^ Compound **1M**, **2M** and **3M** absorb at 368 nm (*ϵ*=62800 M^−1^ cm^−1^), 357 nm (*ϵ*=51000 M^−1^ cm^−1^) and 510 nm (*ϵ*=33800 M^−1^ cm^−1^), respectively, while their emissions occur at 427 nm, 399 nm, and 578 nm, respectively. For the dicationic compounds, the absorption and emission spectra in water are almost identical to those in MeCN. The S_1_←S_0_ absorption for all of these compounds are mainly HOMO‐to‐LUMO transitions, as confirmed by TD‐DFT calculations (see below). Solid state emissions for **3** (590 nm) and **3M** (649 nm) are bathochromically shifted compared to their emission in solution. All of the neutral compounds (**1**–**3**) show very small Stokes shifts (360–700 cm^−1^), while the dications (**1M**–**3M**) show larger Stokes shifts (2300–3870 cm^−1^), which are, nevertheless, smaller than those of the analogous pyrene compounds.^[16]^ Compound **3** has the highest quantum yield of 0.93 in MeCN, and essentially unity (1.0) in CH_2_Cl_2_. Compound **1** has a moderate quantum yield of 0.73, while **2** has a very small one (0.03). Upon methylation, the quantum yield of **1M** drops to 0.30 and for **3M** to 0.66, while it increases for **2M** to 0.52 in MeCN. All of the neutral, as well as the dicationic compounds, have short‐lived excited states, for example, <0.5 ns for **2** and 3.9 ns for **3M**. All of the dicationic compounds exhibit bathochromically shifted emissions compared to their respective neutral compounds. The presence of a fluorinated moiety in **2M** might cause the hypsochromic shift of the emission maximum of 1470 cm^−1^ compared to **1M** in aqueous buffer. The emission spectra in various solvents (MeCN and water for the dications, and MeCN and toluene for the neutral compounds) (Table 1) are similar indicating a lack of solvatochromism. The neutral compounds (except **2**) possess much higher quantum yields compared to their cationic analogues. All of the compounds possess very low quantum yields in the solid state, likely due to the presence of strong π‐stacking interactions.

**Table 1:** Summary of photophysical data for compounds **1**–**3** and **1M**–**3M**.

Cpd	Solvent	*λ*_abs_ [nm]	*ϵ* [M^−1^ cm^−1^]	*λ*_em_ [nm]	Stokes shift [cm^−1^]	*Φ*	*τ* [ns]^[a]^	*τ*_avg_ [ns]	*τ*_0_ [ns]^[b]^	*k*_r_ 10^6^ [s^−1^]	*k*_nr_ 10^6^ [s^−1^]
**1**	MeCN	316 335	53200 37100	342 357 369	700	0.73	0.5^[c]^	–	0.7	1428	540
**1**	Toluene	320 339		347 363 375	680	0.61	<1.0^[d]^	–			
**1**	Solid			429		0.12	1.9 (92.4) 6.4 (7.6)^[d]^	2.2			
**2**	MeCN	314 333	49400 34200	340 358 366	620	0.03	<0.5^[c]^	–			
**2**	Solid			378 386 411 443		0.17					
**3**	MeCN	434 464	19100 20900	472 503 535	365	0.93	3.5 ^*d*^	–	3.8	263	20
**3**	Toluene	439 465		477 510 542	540	0.88	3.2^[d]^	–	3.6	277	37
**3**	Solid			516 548 590 623		0.01	1.3 (71.4) 3.1 (24.6) 13.0 (4)^[d]^	2.2			
**1M**	MeCN	354 368	61000 62800	427	3750	0.30	1.3 ^*c*^	–	4.3	232	538
**1M**	Aq. buffer	349 365	63500	425	3870						
**1M**	Solid			480		0.13	2.0 (25.2) 4.9 (65.3) 10.0 (9.5)^[d]^	4.7	36.1	28	163
**2M**	MeCN	338 357	58000 51000	399	2950	0.52	0.8^[c]^	–	1.5	666	600
**2M**	Aq. buffer	339 357	57500	400	3000						
**2M**	Solid			414 448		0.16					
**3M**	MeCN	483 510	31500 33800	578	2300	0.66	3.9^[d]^	–	5.9	169	87
**3M**	MeOH	483 512		577	2200	0.57	3.5^[d]^	–	6.1	164	123
**3M**	Water	498 520		578	1930	0.64	0.9^[d]^	–	1.4	714	400
**3M**	Solid			649		0.02	0.8 (54.5) 2.1 (42.5) 8.3 (4)^[d]^	1.66			
**3M’**	MeCN	485 510	32200 35700	578	2300	0.64	3.9	–	6.1	164	92

[a] Pre‐exponential factors *B*_n_ scaled to 100 and given in parentheses. [b] Pure radiative lifetime, *τ*_0_=*τ*/*Φ*; *k*_r_=1/*τ*_0_; *k*_nr_=(1−*Φ*)/*τ* . [c] Measured with an Edinburgh Instruments FLS980 spectrophotometer. [d] Measured with an Edinburgh instruments FLS920 spectrophotometer.

**Figure 3:**
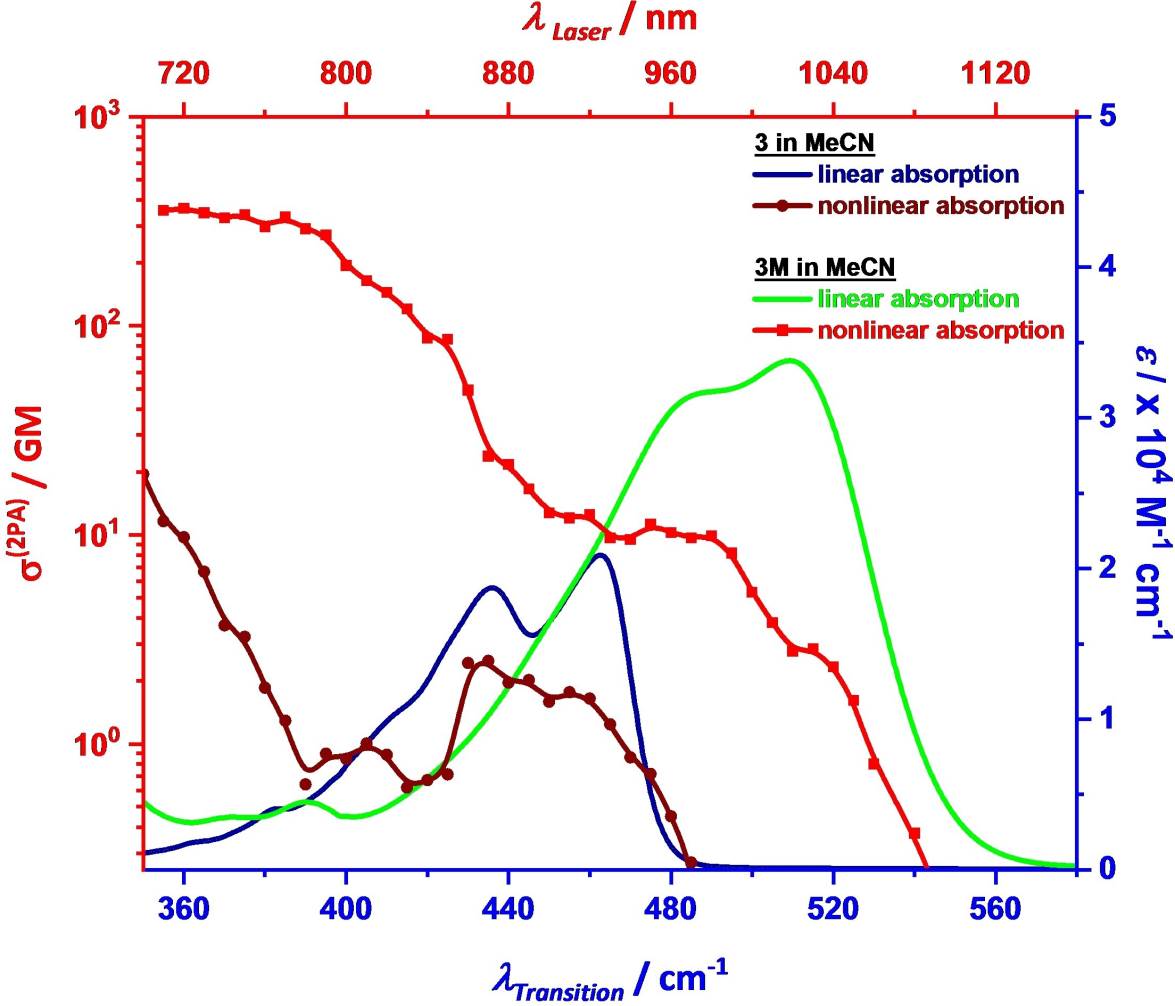
A comparison of the linear (solid lines) and two‐photon absorption on a log scale (lines with dots) spectra of compounds **3** (dark colour) and **3M** (light colour) in MeCN.

Anthracene^[40]^ and π‐conjugated anthracene derivatives^[41]^ are well explored compounds that exhibit enhanced two‐photon absorption cross sections. Τhe two‐photon absorption (2PA) spectra of both **3** and **3M** compounds in MeCN were measured via two‐photon excited fluorescence spectroscopy (Figure 3). Both compounds possess a centre of inversion, and thus the electronic transition selection rules between one‐ and two‐photon transitions are complementary. The least intense 2PA band with a cross‐section <10 GM is observed at the energetic position where the 1PA spectra exhibits the lowest electronic transition (S_1_) and its subsidiary vibronic shoulder for both compounds. These electronically forbidden two‐photon transitions are weakly allowed due to vibronic coupling to asymmetric vibrations of the first one‐photon allowed excited state (Herzberg‐Teller effect).^[42]^ The next 2PA band with maximum 2PA cross‐sections reaching 370 GM for **3M** and ca. 20 GM for **3**, respectively, originates from the higher two‐photon allowed S_2_ state which is one‐photon forbidden.[^40c^, ^40d^] The dicationic compound **3M** exhibits a much larger 2PA cross‐section than the neutral compound **3** throughout the spectral range. We can also compare the 2PA properties of **3M** with those of triarylamine^[43]^ or dialkylaminophenyl^[41a]^ substituted 9,10‐dialkynylanthracene chromophores with the popular donor‐acceptor‐donor motif. For the triarylamine derivative the one‐photon absorption spectral distribution is quite similar to **3M**, but with a higher extinction coefficient for the transition to S_1_. The 2PA maximum at 842 nm (421 nm for 1PA) is also similar, but possesses a distinct maximum of ca. 400 GM. Thus, while in **3M** the central anthracene acts as the donor in a quadrupolar acceptor‐donor‐acceptor chromophore, it serves as an acceptor in the topologically similar triarylamine‐anthracene conjugate with donor‐acceptor‐donor structure but both chromophores show quite similar 2PA properties.

## Theoretical calculations

In order to rationalize the observed spectroscopic and electrochemical (see below) properties, in‐depth density functional‐theory (DFT) and time‐dependent density functional‐theory (TD‐DFT) calculations were carried out. Geometry optimizations and vibrational frequencies calculations were first performed at the PBE0/GD3BJ level using the 6–31G* basis set with the Gaussian09 program (see computational details in the Experimental section). Then, TD‐DFT computations of the UV‐Vis, as well as the circular dichroism (CD) spectra were carried out at the same level of theory, and also using the CAM‐B3LYP functional for comparison. Selected experimentally determined (crystallographic) and theoretically optimized structural parameters are listed in Table S2. Overall, the optimized structural parameters for all the dications (**1M**, **2M**, **3M**) match very well with the crystallographically determined parameters. Small deviations (2°–6°) between dihedral angles are observed, but were expected as the experimental (X‐ray diffraction) ones were measured in the solid state, whereas the theoretical ones were computed considering the molecules in solution (MeCN). The computed ground state structures of the different compounds are planar, but computations show that slight rotations (either inner or outer twist) of one cycle with respect to the others around the alkynyl bonds (see Figure S21 for **1M** for example) require little energy up to ca. 30° (a few tenths of an eV, see Figure S22) indicating that rotamers might well coexist at room temperature in solution.

**Figure 4:**
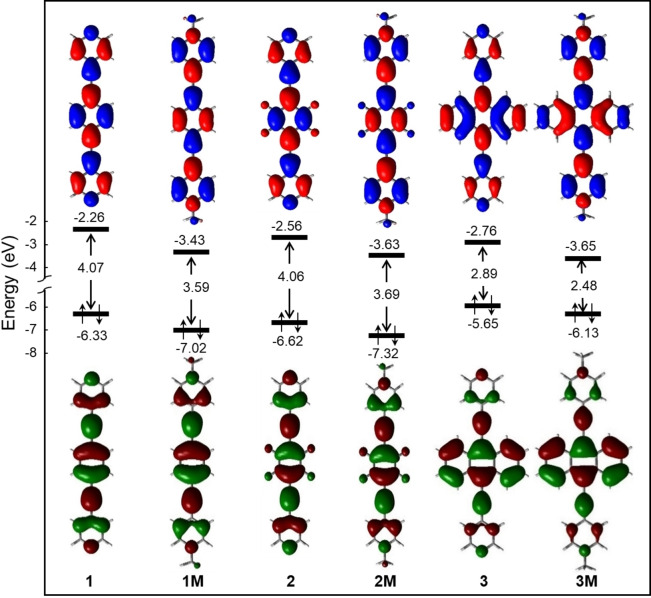
Energies (eV) and nodal representation of the HOMO and LUMO of the bis‐(4’‐pyridylethynyl)arenes **1**–**3** and their bis‐*N*‐methylpyridinium compounds **1M**–**3M** calculated at the PBE0‐GD3BJ/6‐31G* level (isocontour value: ±0.03 [e.bohr^−3^]^1/2^).

The frontier molecular orbitals (FMOs) of the different bis‐(4’‐ethynylpyridyl)arenes **1**–**3** and their *N*‐methylpyridinium compounds **1M**–**3M** are displayed in Figure 4. As expected, they are of π‐type and highly similar in character and heavily delocalized over the bis‐(4’‐pyridylethynyl)arene backbone. Though a strict comparison between neutral and dicationic species cannot be done, the HOMO‐LUMO gaps are smaller for the dicationic than the neutral species. This suggests that the dicationic compounds should absorb light at lower energies, as was observed experimentally (see above). Comparing compounds **1** and **2**, the effect of the fluorine attached to the phenyl ring lowers both the HOMO and LUMO energies; however, the HOMO‐LUMO gap remains almost unchanged. Compound **3** exhibits the highest HOMO energy indicating the highest donating character relative to other compounds. The smallest HOMO‐LUMO gaps are observed for compounds **3** and **3M**, in line with experiment, with absorption occurring at lower energies (see above).

The results of the TD‐DFT computations of the UV‐Vis absorption spectra are given in Table 2 and Figure S23, where they are compared to the observed absorption values. As can be seen, there is a very good agreement between the computed wavelengths using the CAM‐B3LYP functional and the experimentally measured values, whereas the PBE0 functional leads to values somewhat overestimated, but the computed absorption wavelengths follow the same trend as the experimental data. These intense absorptions are almost exclusively due to HOMO‐LUMO transitions in all cases. The two peaks observed experimentally correspond to the vibronic structure of the electronic absorption band. The simulated UV‐Vis spectra of different rotamers displayed in Figures S24–S26 indicate that they are comparable overall with, as expected, a slight blue shift upon deviation from planarity (see the computed maximum absorption wavelengths for the rotamers of **1M**‐**R** in Table S4, for example).

**Table 2:** Experimental and PBE0‐GD3BJ/6‐31G* and CAM‐B3LYP‐GD3BJ/6‐31G* calculated maximum absorption wavelengths (*λ*, nm) in MeCN for **1**, **1M**, **2**, **2M**, **3**, and **3M**. *f* Values are oscillator strengths while vertical excited state number (S_n_) are given in parentheses.

Cpd	*λ*_exp_ (MeCN)		*λ*_cal_	*f* (S_*n*_)	Main electronic transition [weight%]
**1**	316, 335	PBE0	355	1.99 (S_1_)	HOMO→LUMO (+98 %)
		CAM‐B3LYP	327	1.98 (S_1_)	HOMO→LUMO (+92 %)
**1M**	354, 368	PBE0	428	2.35 (S_1_)	HOMO→LUMO (+98 %)
		CAM‐B3LYP	383	2.41 (S_1_)	HOMO→LUMO (+90 %)
**2**	314, 333	PBE0	355	1.97 (S_1_)	HOMO→LUMO (+98 %)
		CAM‐B3LYP	330	1.94 (S_1_)	HOMO→LUMO (+92 %)
**2M**	338, 357	PBE0	396	2.10 (S_1_)	HOMO→LUMO (+98 %)
		CAM‐B3LYP	358	2.15 (S_1_)	HOMO→LUMO (+90 %)
**3**	434, 464	PBE0	507	0.92 (S_1_)	HOMO→LUMO (+100 %)
		CAM‐B3LYP	463	0.99 (S_1_)	HOMO→LUMO (+98 %)
**3M**	483, 510	PBE0	606	1.03 (S_1_)	HOMO→LUMO (+100 %)
		CAM‐B3LYP	528	1.20 (S_1_)	HOMO→LUMO (+98 %)

**Figure 5:**
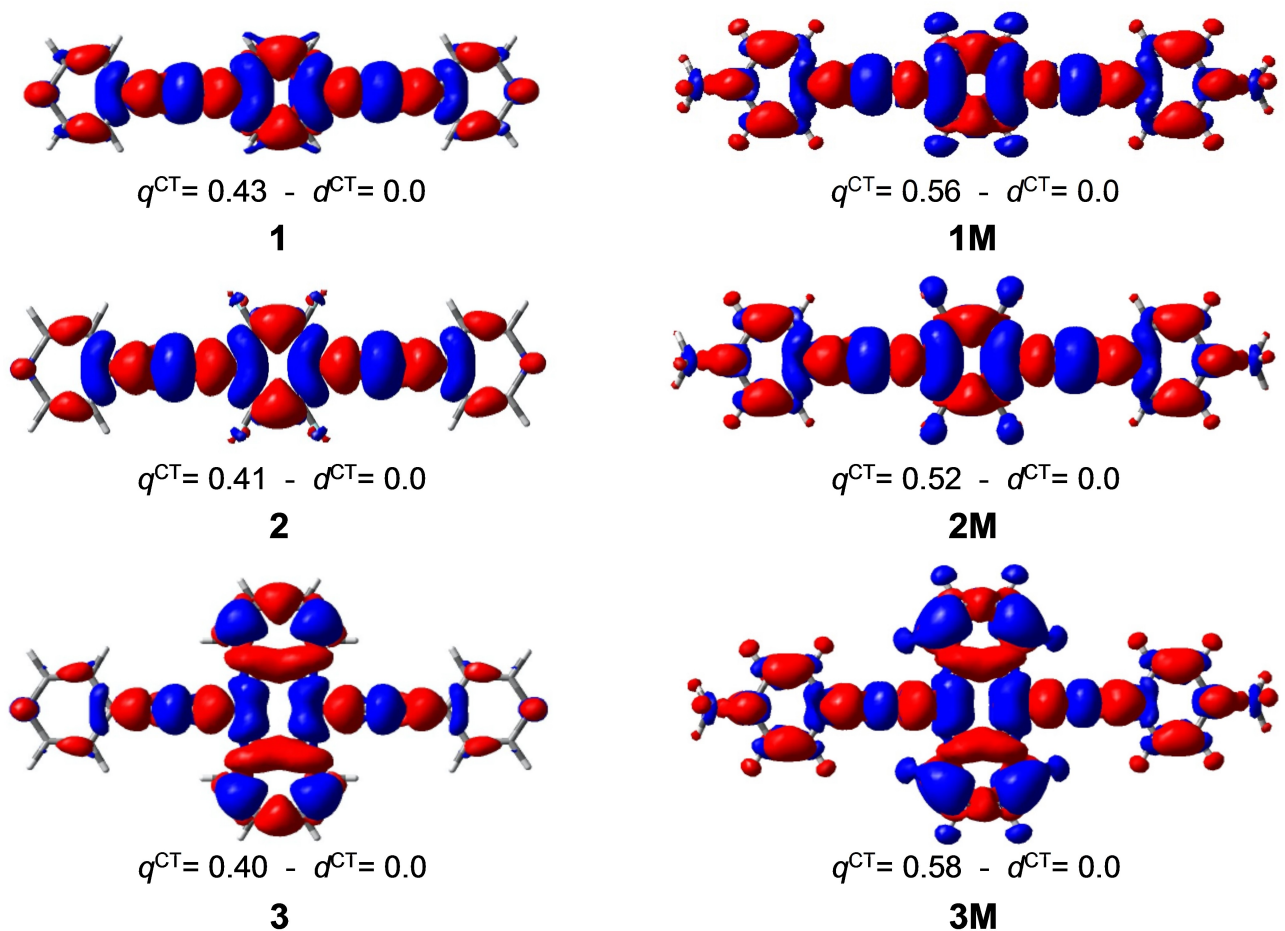
Plots of the difference *ρ* ^S1^(*r*) ‐ *ρ* ^S0^(*r*) of the CAM‐B3LYP electron densities of S_1_ and S_0_ states of the bis‐(4’‐pyridylethynyl)arenes **1**–**3** (left) and their bis‐*N*‐methylpyridinium compounds **1M**–**3M** (right). The red and blue colours indicate an increase and decrease of electron density upon excitation, respectively (isocontour value=0.0004 e.bohr^−3^). *q* ^CT^ (e) and *d* ^CT^ (Å) correspond to the transferred charge and the distance between the positive and the negative centroids of charge, respectively.

The difference density plots between the vertical S_1_ state and the ground state S_0_ are shown in Figure 5 for the bis‐(4’‐pyridylethynyl)arenes **1**–**3** and their N‐methylpyridinium compounds **1M**–**3M**. The amount of transferred charge *q* ^CT^ is higher for the methylated species, the difference being the highest for **3** and **3M** compounds, i. e., 0.40 electron (e) for **3** (Figure 5) vs. 0.58 e for **3M**. This charge transfer upon excitation mostly occurs from the center of the molecules towards the remote parts. Note that the *d* ^CT^ distance between the centroids of the positive and negative charges is equal to zero for all compounds due to their symmetry.

The emission wavelengths *λ*_em_ and corresponding oscillator strengths *f*_em_ were computed as the energy difference between the relaxed S_1_ state and the ground state (with the S_1_ geometry). The geometry optimizations of the S_1_ states were carried out at the PBE0‐GD3BJ/6‐31G* level in MeCN solvent. The results in Table S3 indicate that the CAM‐B3LYP computed *λ*_em_ values are in very good agreement with the experimental data, whereas those obtained with the PBE0 functional are largely overestimated, in line with the computed absorption wavelengths (Figure S23).

## Electrochemistry

**Figure 6:**
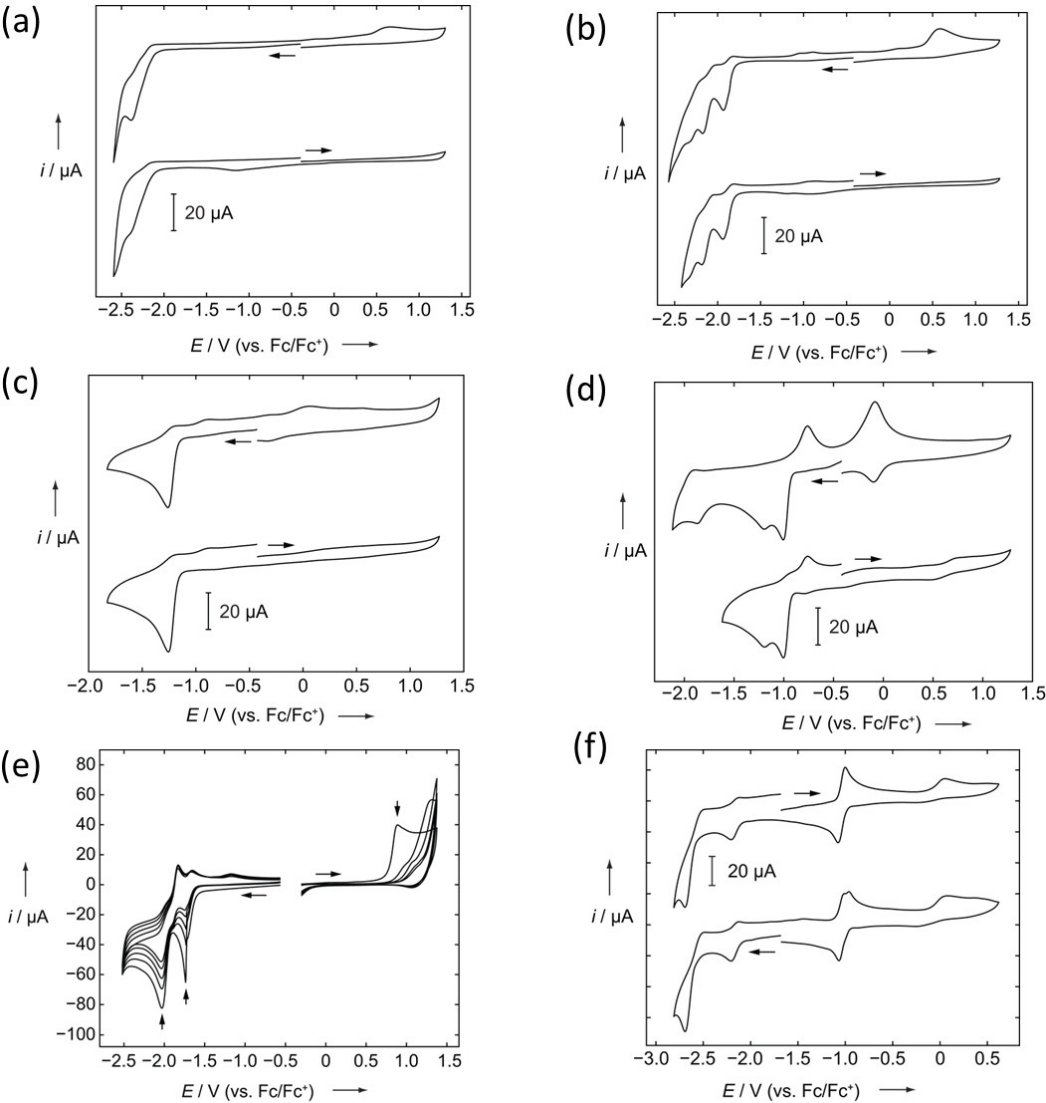
Cyclic voltammograms of a) **1** and b) **2** in CH_2_Cl_2_/0.1 M [*n*‐Bu_4_N][PF_6_]; c) **1M** and d) **2M** in MeCN/0.1 M [*n*‐Bu_4_N][PF_6_]; e) multiple scans of **3** in CH_2_Cl_2_/0.1 M [*n*‐Bu_4_N][PF_6_] (vertical arrows show the decrease of peak size with repeated scans), f) **3M** in DMF/0.1 M [*n*‐Bu_4_N][PF_6_]. A scan rate of 250 mV s^−1^ was applied for all the measurements.

Cyclic voltammetry was performed on the neutral and methylated compounds to investigate their electrochemical properties. The voltammograms were measured at a scan rate of 250 mV s^−1^ with 0.1 M [*n*‐Bu_4_N][PF_6_] relative to the Fc/Fc^+^ couple. The measurements were carried out in CH_2_Cl_2_ for the neutral compounds (**1**, **2**, **3**). MeCN was used for the dicationic compounds (**1M**, **2M**), whereas DMF was used for **3M** (Figure 6). Compounds **1**, **1M**, **2**, **2M** show only irreversible reduction processes and no observable oxidation events up to the respective solvent limit, when the initial scan direction is anodic. The fluorinated derivatives (**2** and **2M**) show multiple reduction events and are more readily reduced (i. e., they have lower reduction potentials) than the nonfluorinated analogues (**1** and **1M**). The cationic derivatives (**1M** and **2M**) are more easily reduced than their neutral counterparts. The reductions for **1** and **1M** occur at *E*_pc_=−2.38 V (corresponding oxidation: *E*_pa_=0.65 V), and −1.25 V, respectively. Similarly, in the case of **2** and **2M**, the formal potentials for the reductions were determined to be *E*_pc_=−1.99 V and −2.20 V with corresponding oxidation, *E*_pa_=0.58 V (for **2**), and *E*_pc_=−1.01 V, −1.12 V (corresponding oxidations: *E*_pa_=−0.77 V, −0.07 V), and −1.86 V (for **2M**). Potential scans for **3** were initiated at the rest potential (ca. −0.5 V) in CH_2_Cl_2_/0.1 M [*n*‐Bu_4_N][PF_6_], and reversed at the anodic and cathodic potential limit, respectively. Multiple scans are shown in the positive and negative direction, with each new cycle starting at the rest potential. Two reduction events were observed to occur at *E*_pc_=−1.74 V, *E*_pc_=−2.03 V, whereas one oxidation event occurred at *E*_pa_=0.78 V in the anodic direction. Additional cycles led to disappearance of the oxidation peak and a gradual decrease in reduction peak currents. Compound **3M** exhibits three reduction events at *E*_1/2_=−1.04 V, *E*_pc_=−2.16 V, and −2.69 V, and an oxidation event at *E*_pa_=0.05 V. In contrast to **1M** and **2M**, but in line with related dicationic viologen compounds,^[1]^ **3M** exhibits a reversible reduction event at around *E*_1/2_=−1.04 V, which can best be ascribed either to two closely spaced one‐electron reductions or a two‐electron single step reduction of the pyridinium rings.[^11a^, ^16^] Two additional reductions at −2.16 V, and −2.69 V can be attributed to the stepwise one‐electron reduction of the anthracene unit (Figure 6f).^[44]^

## Study of interactions with DNAs and RNAs

**Figure 7:**
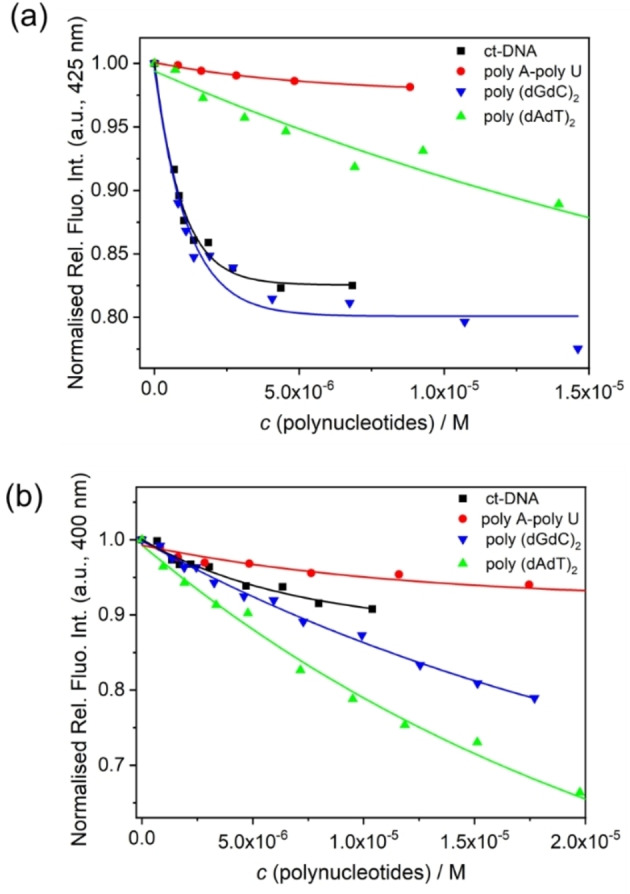
Changes in fluorescence intensity of a) **1M** (*c*=5×10^−7^ M) upon addition of polynucleotides; and b) **2M** (*c*=5×10^−7^ M) upon addition of polynucleotides, at pH 7.0, sodium cacodylate buffer, *I*=0.05 M. The lines denote non‐linear fitting of data to the Scatchard equation.^[48]^

Compounds **1M**‐**3M** are moderately soluble in water. Thus, their stock solutions were prepared in DMSO (*c*=5×10^−3^ M), and aqueous solutions were prepared from them prior to each experiment. Stock solutions were stable for longer periods when stored at +4 °C. Upon heating the solutions, the UV‐Vis spectra of **1M** and **2M** showed only negligible decreases with temperature. However, **3M** showed a small increase in the UV range. Interactions of **3M** with ds‐DNAs, reported recently,^[39]^ revealed an intercalative binding mode, characterised by typical thermal denaturation stabilisation of ds‐DNAs, strong hypochromic and bathochromic effects, as well as fluorescence emission changes and binding constants in the log *Ks*=6–7 M^−1^ range, which were further corroborated in our study, performed under slightly different conditions (sodium cacodylate buffer instead of Tris buffer). Addition of any ds‐DNA or ds‐RNA to **1M** and **2M** caused only minor hypochromic changes, quenched by 5 %–25 % in their UV‐Vis spectra. The fluorescence from **1M** and **2M** was weakly quenched upon addition of various ds‐DNAs or ds‐RNAs (Figure 7), with the non‐fluorinated analogue **1M** showing somewhat better selectivity between various base pair sequences in comparison to the tetrafluoro‐analogue **2M**.

**Figure 8:**
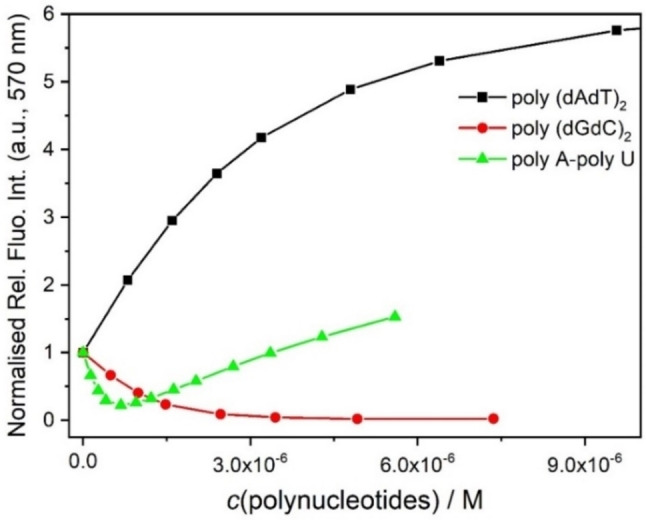
Dependence of **3M** (*c*=5×10^−7^ M, *λ*_ex_=500 nm) emission intensity at *λ*_max_=570 nm upon titration with poly(dAdT)_2_ (–)_,_ poly(dGdC)_2_ (–) and polyA‐poly U (–), at pH 7.0, sodium cacodylate buffer, *I*=0.05 M. Red line denotes non‐linear fitting of data to the Scatchard equation.^[48]^

Intriguingly, **3M** showed an opposite fluorescence response for GC‐DNA (Figure 8, total quenching) in comparison to AT‐DNA (Figure 8, strong increase), in accordance with reported data.^[39]^ The same type of response was previously observed for proflavine and some pyrene derivatives, and was attributed to the more pronounced electron‐donating properties of guanine compared to other nucleobases.^[45]^ Under the same titration conditions with AU‐RNA, **3M** showed an emission quenching at an excess of dye over RNA binding sites (ratio *r*_[**3M**]/[AU‐RNA]_ >0.5), which switched to an increase of emission intensity at an excess of RNA (*r*<0.2). In contrast to ds‐DNAs, ds‐RNA adopts an A‐helical structure, characterized by a very broad and shallow minor groove (Supporting Information, Table S5), which, due to exposure to bulk water, is not suitable for small molecules binding. However, small molecules fit nicely into the ds‐RNA deep, narrow (Supporting Information, Table S5) and, consequently, hydrophobic major groove, in which, upon expulsion of water, the dye can additionally form H‐bonding and aromatic interactions.^[46]^ When there is an excess of the small molecule with respect to the RNA‐binding site, sometimes small molecules form stacked dimers within the polynucleotide binding site (very often noticed for cyanine dyes^[46]^ and recently for a 2,7‐bis(N‐methyl‐4’‐pyridinium)pyrene derivative^[16]^), whereby, upon addition of excess of RNA, small molecules eventually redistribute to separate binding sites. Similar behaviour was observed for **3M**, which, when in excess with respect to RNA (*r*[**3M**]/ [RNA] >0.5), forms a self‐stacked dimer with quenched emission due to aromatic stacking interactions between anthracenes and, upon adding more RNA (*r*<0.2), switched to single **3M**‐molecules interacting with RNA independently, resulting in the emission increase. Such changes can be attributed to the deep major groove of RNA,^[47]^ which can accommodate dimers of **3M** at *r*>0.5. Fluorimetric titration data were further processed by means of non‐linear fitting to the Scatchard equation^[48]^ to determine the binding constants (Table 3), which offer valuable information about their binding strength with DNA/RNA.

**Table 3:** Binding constants (log *K*_s_/*M* ^−1^)^[a]^ of **1M**, **2M** and **3M** complexes with ds‐polynucleotides calculated by processing fluorimetric titrations (*c*=5×10^−7^ M), at pH=7, sodium cacodylate buffer, *I*=0.05 M.

	ct‐DNA	p(dAdT)_2_	p(dGdC)_2_	pApU
**1M**	6.0	4.5	5.9	5.3
**2M**	5.9	5.2	5.1	5.0
**3M**	6.3	6.6	7.0^[b]^	5.3

[a] Processing of titration data by means of Scatchard equation^[48]^ gave values of the ratio *n*[bound dye]/ [polynucleotide]=0.1 and 0.2. For easier comparison, all log *K*_s_ values (/M^−1^) were re‐calculated for fixed *n*=0.1. Correlation coefficients were >0.99 for all calculated values of log *K_s_*. [b] In agreement with reported data.^[39]^

**Figure 9:**
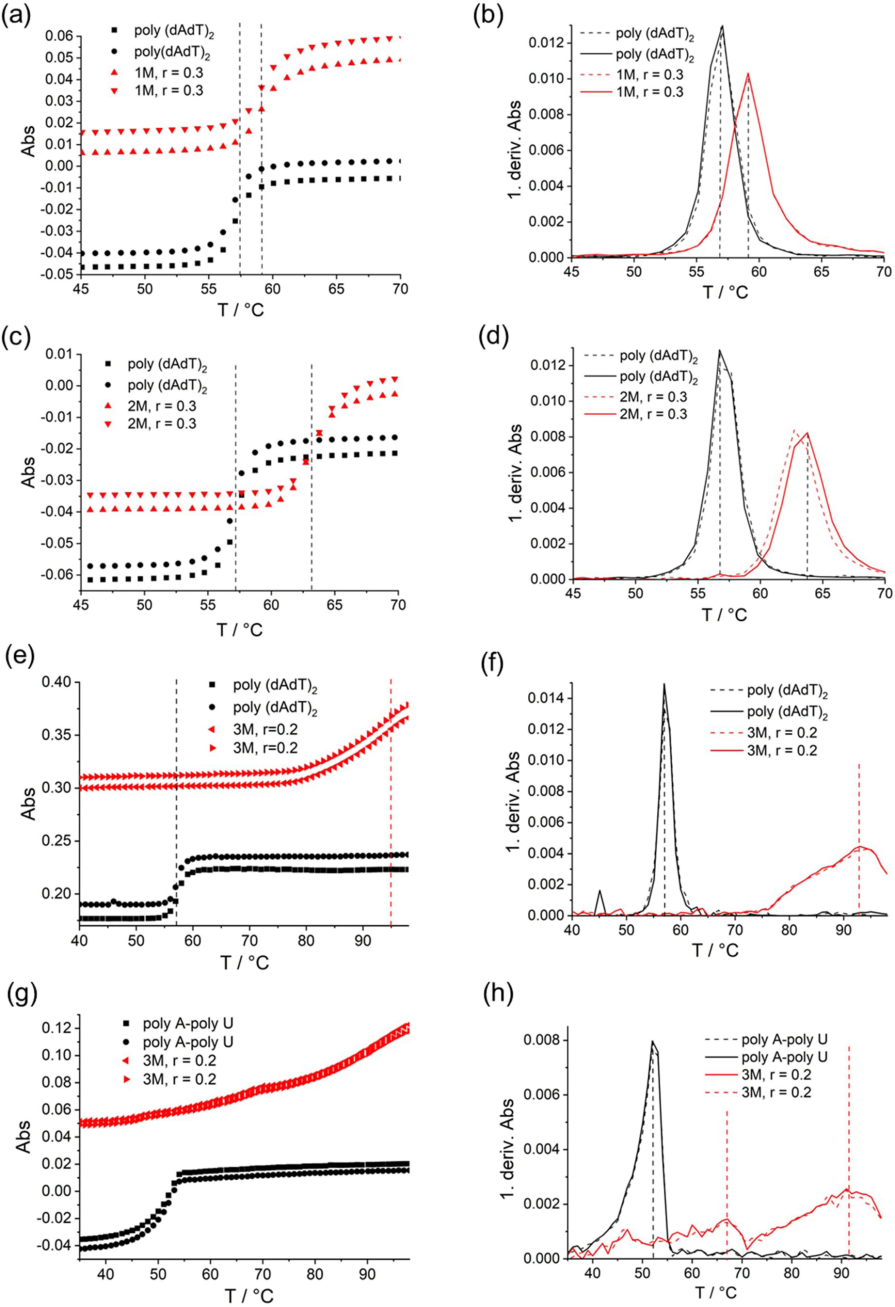
Thermal denaturation of p(dAdT)_2_ (*c*=2×10^−5^ M, 260 nm) and first derivative of absorbance on temperature upon addition r=0.3 ([compound/ [polynucleotide]) of **1M** (a, b); and **2M** (c, d) in aqueous buffer (sodium cacodylate, pH=7, *I*=0.05 M). Thermal denaturation of p(dAdT)_2_ (*c*=2×10^−5^ M, 260 nm) (e, f) and pApU (*c*=2×10^−5^ M, 260 nm) (g, h); and first derivative of absorbance on temperature upon addition *r*=0.2 ([compound]/ [polynucleotide]) of **3M** in aqueous buffer (sodium cacodylate, pH=7, *I*=0.05 M).

Thermal denaturation experiments of ds‐DNA/RNA were performed, noting that addition of **1M** caused only minor stabilisation of ds‐DNA but had no effect on ds‐RNA (Table 4, Figure 9a, b), which can be attributed to the more appropriate size and shape of the DNA minor groove for binding of small molecules.^[48]^ Surprisingly, addition of **2M** resulted in much stronger stabilisation for poly‐(dAdT)_2_ (Table 4, Figure 9c, d), which suggests that the central fluorinated core of **2M** forms additional binding interactions within the minor groove, but these are selective for homogeneous AT‐DNA sequences, as mixed sequence ct‐DNA (48 % GC‐base‐pairs) was only marginally stabilised by **2M**. However, the impact of **3M** on the stabilization of ds‐polynucleotides was very strong for all DNAs/RNAs investigated (Table 4, Figure 9e–h), indicating intercalation as a dominant binding mode.^[49]^ This was further corroborated by an increase of viscosity of ds‐DNA solution upon addition of **3M**[^50^, ^51^, ^52^] (see Supporting Information for details).

**Table 4:** [a] Δ*T_m_* (°C) for different ratios r [compd.]/[polynucleotide] of **1M**, **2M** and **3M** added to polynucleotides.

	r	ct‐DNA	p(dAdT)_2_	pApU
**1M**	0.2	2.0	–	–
	0.3	2.0	2.0	0
**2M**	0.2	1.0	–	–
	0.3	1.0	7.0	1.0
**3M**	0.05	3	–	–
	0.1	16	13.0; 33.0^[b]^	4.0; 16.0; 39.0^[b]^
	0.2	–	37	39.0

[a] Error in *▵T*_m_=±0.5 °C; [b] biphasic transitions: all transitions are attributed to an uneven distribution of compound at polynucleotide regions at low ratio *r*.

## Circular dichroism (CD) experiments

In order to obtain additional insight into the changes of the polynucleotide properties induced by small molecule binding, we chose highly sensitive CD spectroscopy to observe conformational changes in the secondary structure of polynucleotides.^[53]^ In addition, achiral small molecules can acquire induced CD (ICD) upon binding to polynucleotides, which can provide useful information regarding modes of interaction.[^47^, ^53^, ^54^] It should be noted that compounds **1M**–**3M** are achiral, and therefore do not possess intrinsic CD spectra.

**Figure 10:**
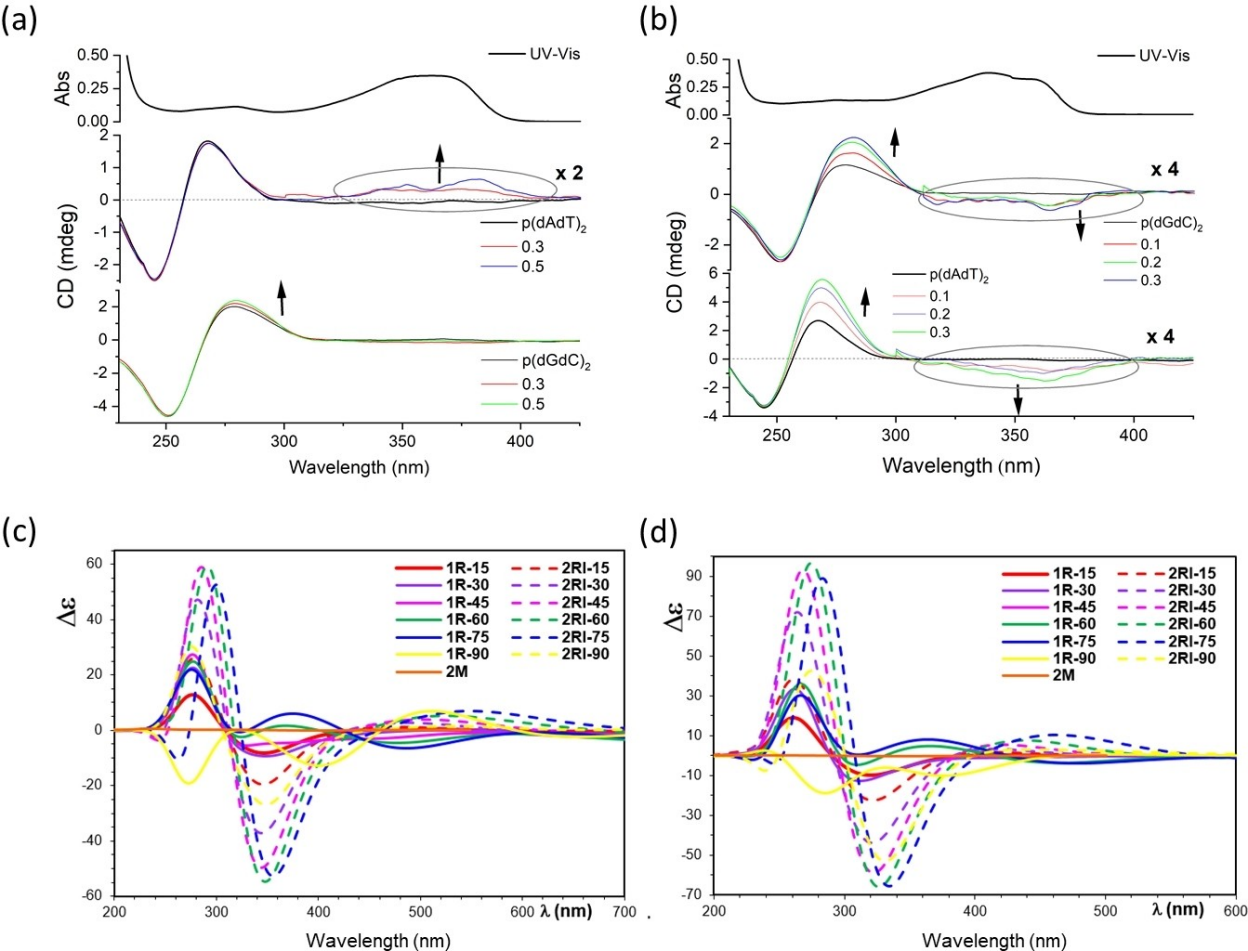
CD titrations of poly (dG‐dC)_2_ and poly (dA‐dT)_2_ (*c*=2×10^−5^ M) at various ratios *r*[dye]/[polynucleotide] with: a) **1M**, and b) **2M**. The upper spectra in each case (black line) are UV‐Vis spectra of **1M** and **2M**, respectively, at concentrations corresponding to *r*=0.3. These were measured in aqueous buffer (sodium cacodylate) at pH=7.0, *I*=0.05 M. c) PBE0 and d) CAM‐B3LYP simulated CD spectra (Δ*ϵ*, 10^−40^ esu^2^ cm^2^) of **2M** rotamers in water labelled **1R‐d**, where d is the dihedral angle of the rotated pyridinium ring relatively to the planar core of the molecule, and **2RI‐d** when the two terminal pyridinium rings are rotated with respect to the central ring.

Only minor change in the CD band of ds‐DNA/RNA at 245 nm upon addition of **1M** and **2M** indicate that the helicity of the polynucleotide double helix was not significantly perturbed (Figure 10). Some changes can be observed at 260–280 nm (commonly attributed to nucleobases organized in the helix[^47^, ^53^, ^54^]), but only for the tetrafluoro analogue **2M**. However, as **2M** absorbs in that region, it is not possible to comment with certainty on whether the changes are caused by re‐arrangement of the nucleobases or by an induced (I)CD effect of the small molecule chromophore (**2M**). Regarding the changes at wavelengths longer than 300 nm, where **1M** and **2M** absorb, the observed novel bands can be attributed to the ICD effects of well‐oriented chromophores with respect to the chiral axis of the polynucleotide. Addition of either of the dicationic compounds did not change the CD spectrum of RNA (poly A ‐ poly U) significantly, which is likely due to the large size of the RNA grooves (Supporting Information, Table S5), which allows heterogeneous orientation of small molecules.

The observed difference in ICD bands for **1M** and **2M** (Figure 10) could be attributed to the structural difference between compounds in the central phenyl core. Only **1M** exhibited a weak positive ICD band in 330–450 nm range for AT‐DNA, which suggests positioning of the long axis of chromophore along the direction of the DNA minor groove.[^47^, ^54^] However, **2M** resulted in a weak negative ICD band in the same range, proportional to a quite strong increase in the positive CD signal at 280 nm, also sharing an isoelliptic point at ca. 300 nm. Such a change suggests a coupled positive‐negative signal of chromophore **2M** caused by binding to DNA. The calculated CD spectra of **2M** rotamers suggested that **2M**, upon binding to minor groove, was forced into a non‐planar rotamer by the chiral shape of the binding site.

**Figure 11:**
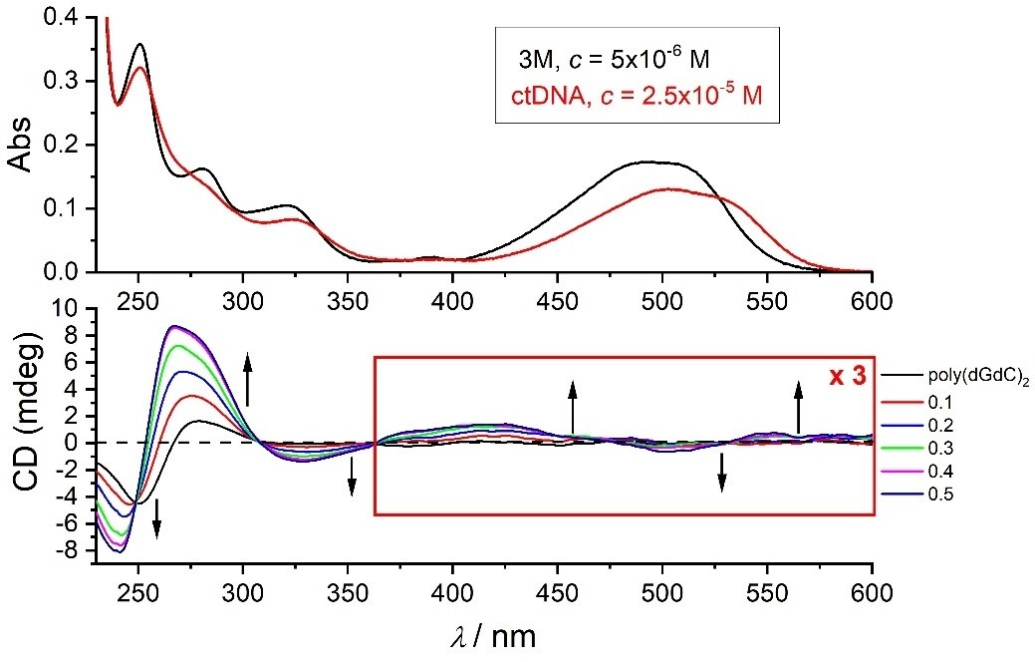
CD titrations of poly (dG‐dC)_2_ (*c*=2×10^−5^ M) at various ratios *r*[dye]/[polynucleotide] with **3M**. Note that the spectrum on top is UV‐Vis titration of **3M** with ct‐DNA (*c*=2×10^−5^ M) (Figure S47 in the Supporting Information), respectively, at concentration *c*=5×10^−6^ M. Measured in aqueous buffer (sodium cacodylate) at pH=7.0, *I*=0.05 M.

**Figure 12:**
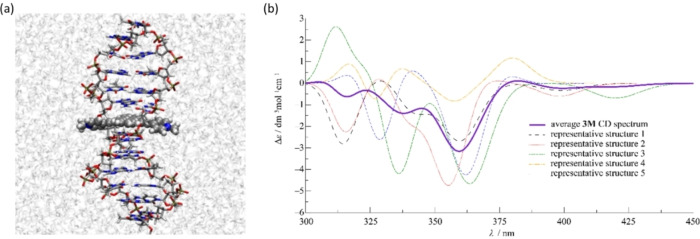
a) Snapshot of the most dominant structure of **3M** intercalated in the model ds‐DNA helix obtained from molecular dynamics (MD) simulation (see Molecular dynamics of **3M**/ds‐DNA complex Section). Water molecules are represented using ghost mode for better visibility (visualized using VMD software^[55]^); b) calculated average CD spectrum of **3M** arising from 20 representative structures found via principal component and clustering analysis of MD simulation of **3M** complex with ds‐DNA (see computation of **3M** CD spectrum section for details). Additionally, CD spectra of 5 most prominent representative structures computed at the CAM‐B3LYP/6‐31G*) level in water solvent are also shown (CD spectrum corresponding to the structure shown in the left side (a) is denoted as representative structure 1).

CD titrations were performed by adding **3M** to DNA/RNA which provided quite unusual and interesting results (Figure 11), mostly consistent with reported results.^[39]^ However, inspired by results observed for **2M**, we suspected that the DNA‐controlled chiral rotamer of **3M** is responsible for the remarkable effect and, therefore, a very detailed CD titration was performed, collecting as many data points as possible. In the 230–300 nm region where DNA/RNA absorb, a strong increase of intensity of CD bands was observed, exceeding the intensity of the intrinsic DNA/RNA CD spectrum by a factor of 2–3. As ds‐DNA/RNA helices cannot increase chirality to such an extent, the observed increase of CD bands can only be attributed to chromophore **3M** becoming chiral upon binding to DNA/RNA. Furthermore, at wavelengths >300 nm, a set of positive and negative ICD bands appeared, and all the changes in the titration were characterised by isoelliptic points, which strongly support only one type of chromophore. This result strongly indicates that upon binding to DNA/RNA, **3M**, similarly to **2M**, is forced into one dominant chiral rotamer. This observation was further supported by calculations of the CD spectra by systematically varying the rotation angle between arene moieties in **3M** (Figure 12).

The spectral signatures of compound **3M** upon binding to ds‐DNA/RNA exhibit quite a rare behaviour, in which the DNA/RNA binding site did not transfer chirality from the ds‐helix to the small molecule by the common induced CD spectrum mechanism;[^47^, ^53^, ^54^] rather, it enforced the otherwise planar (and therefore achiral) small molecule into a chiral rotamer, characterised by an exceptionally strong CD spectrum, which covered all eventual ICD bands (e. g., weak negative ICD band of anthracene in **3M**, expected for intercalation[^47^, ^54^]).

Furthermore, we performed molecular dynamics simulation of **3M** intercalated into a model ds‐DNA strand (11 base pairs, see Molecular dynamics of **3M**/ds‐DNA complex section below for details), which showed that the threading complex of **3M** with ds‐DNA is quite stable, with **3M** being intercalated inside the ds‐DNA groove for the entire duration of the propagated MD simulation (*t*=100 ns; Figure 12a). Moreover, we found that the longer axis of the anthracene moiety remains mostly parallel to the longer axes of adjacent base pairs throughout the MD simulation, in turn governing the positions of cationic pyridinium substituents, which are found at the opposite sides of the ds‐DNA helix, a typical observation for threading intercalation.^[56]^ While the position of the anthracene moiety is well preserved during the MD simulation, the pyridinium substituents have somewhat larger freedom of movement, thus careful analysis and proper sampling of their distribution is necessary to calculate the average CD spectrum of **3M** intercalated into ds‐DNA. Nevertheless, we found that the pyridinium rings of the predominant cluster of **3M** structures favour conformations in which their dihedral angle relative to the anthracene moiety is approximately 30° (Figure 12a). In the emergent average CD spectrum (Figure 12b; see Supporting Information: calculation of **3M** CD spectrum for details) shown in the 300–450 nm range, the negative band appearing at approximately 360 nm, and a positive band arising at about 385 nm agree well with the profile of the experimentally obtained CD spectrum (Figure 11: negative band at ca. 340 nm, and positive at ca. 400 nm). The PBE0/6‐31G* level of quantum‐mechanical theory was utilized to calculate the average CD spectrum of **3M** (see Figure S30 in the Supporting Information), showing qualitative agreement with the results presented in Figure 12b).

Therefore, upon binding to ds‐DNA/RNA, **2M** and especially **3M** showed quite unusual behaviour in their CD spectra. The DNA/RNA binding site did not transfer chirality of the ds‐helix to the small molecule by a typical induced CD spectrum mechanism,[^47^, ^53^, ^54^] rather it enforced the otherwise planar, and thus achiral molecule highly selectively into only one chiral rotameric conformation, characterized by an exceptionally strong CD spectrum, which dominated over all eventual ICD bands (e. g., the weak negative ICD band of anthracene in **3M**, expected for intercalation).[^47^, ^54^]

## Biological activity

**Figure 13:**
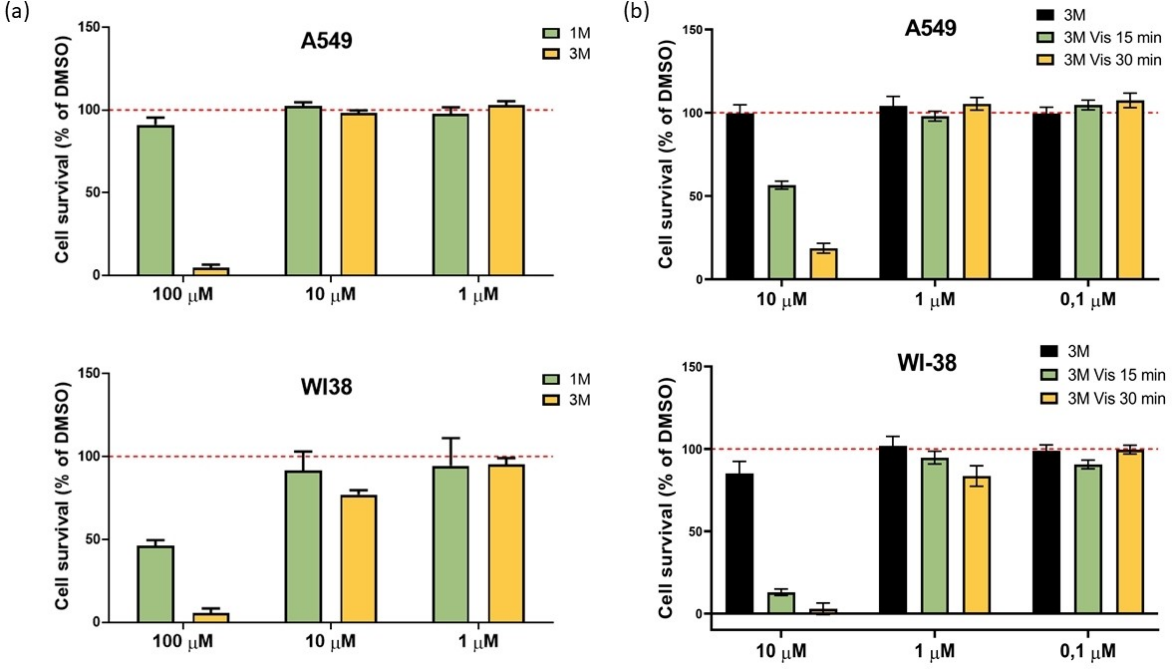
Cell survival of A549 and WI38 cells exposed to: a) **1M** and b) **3M** (with or without exposure to visible light by irradiation in the photoreactor Luzchem, LZC‐ICH2, equipped with eight overhead‐mounted LZC‐vis lamps, 400–700 nm, in total dose 50.6 mW m^−2^, ∼18 cm distance lamp to cell‐plate). Data from 4 replicates are presented as mean±SD, relative to the control samples. Control samples are cells treated with DMSO at the same concentration as the tested compound. Representative data from three independent experiments, which yielded similar results, are shown.

We further tested **1M** and **3M** by the MTT assay (for 72 h,) against two human cell lines,^[57]^ namely epithelial human lung adenocarcinoma A549 (ATCC® CCL‐185™) and human normal lung fibroblast WI‐38 (ATCC® CCL‐75™) cells (Figure 13). Compound **3M** showed significant cell toxicity in both A549 and WI38 cell lines only at the highest concentration (100 μM), whereas compound **1M**, at a concentration of 100 μM, was weakly toxic only for WI‐38 cells. At lower concentrations (10 μM and 1 μM), all compounds did not exhibit cell toxicity in human A549 and WI38 cells, as demonstrated in Figure 13.

**Figure 14:**
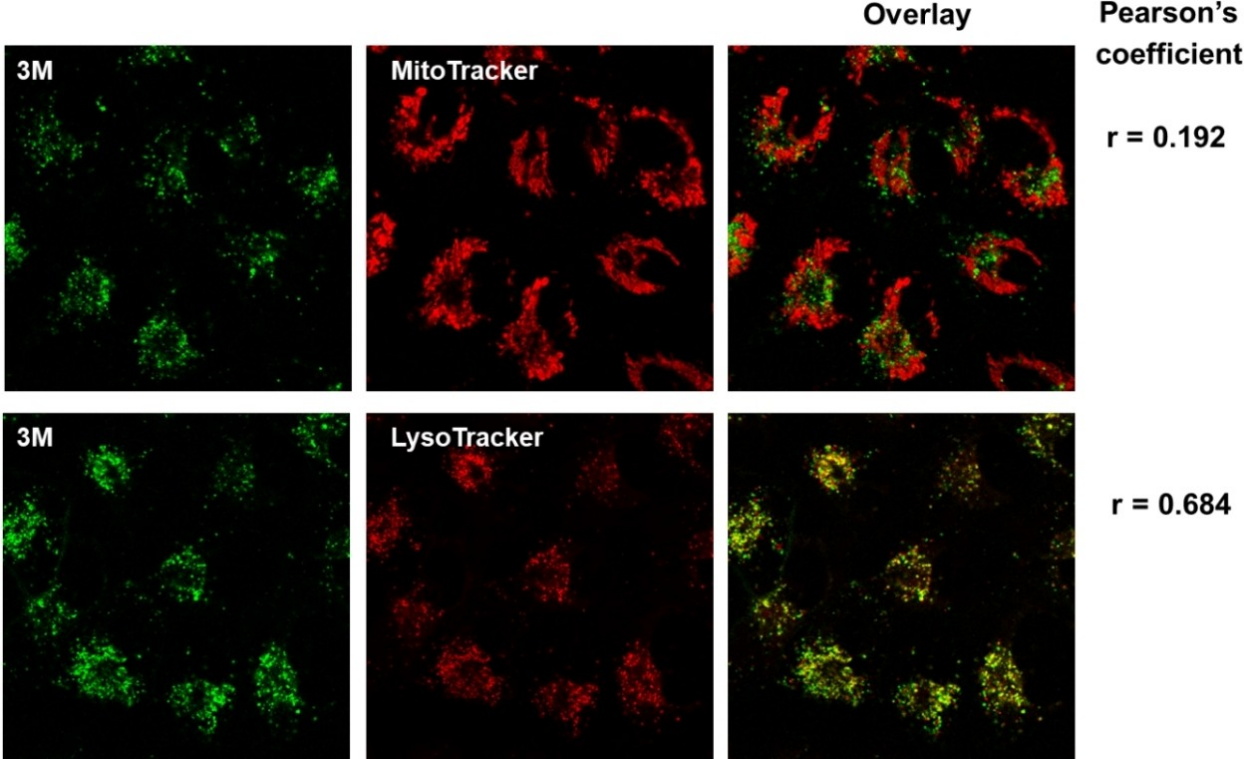
Co‐localization of **3M** (*λ*_ex_=457 nm; *λ*_em_=500–600 nm) with mitochondria and lysosomes observed by confocal microscopy. Cells were treated with 10 μM of **3M** for 90 min at 37 °C and incubated with 100 nM MitoTracker for 20 min or 50 nM LysoTracker for 30 min at 37 °C. Co‐localization was assessed by the Pearson's coefficient. Analysis was done using *ImageJ* software and appropriate *JACoP* plugin.^[59]^

**Figure 15:**
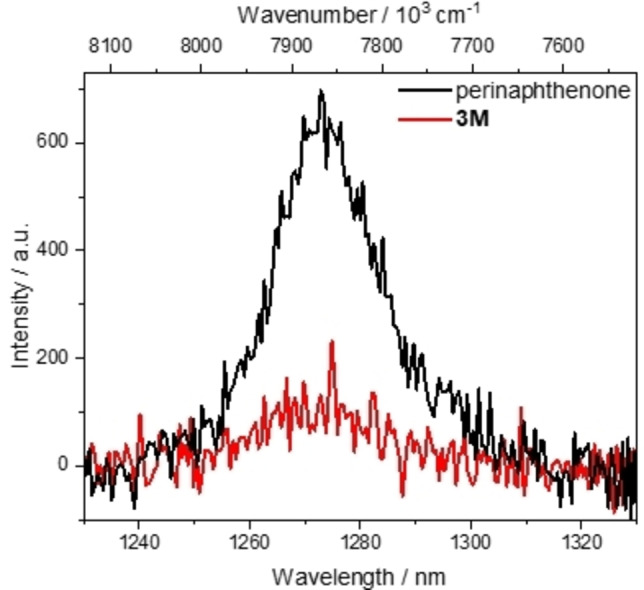
Emission spectrum of singlet oxygen generated from sensitization of a perinaphthenone standard (black) vs. that generated by sensitization by compound **3M** (red) excited at 426 nm in MeCN.

When we exposed A549 cells treated with 10 μM of **3M** to visible light (irradiated by visible light in the photoreactor for 15 and 30 min on the 1^st^, 2^nd^ and 3^rd^ day) we observed a significant increase of cytotoxicity of **3M** (Figure 13b). To understand better the observed photo‐induced toxicity, we performed an experiment on a confocal microscope under the same conditions as described in Figure 14, but exposing several cells to the maximum power of a 457 nm laser for 3 min, and simultaneously monitoring the changes by fluorescence of **3M** and also by a bright field option (see movie in the Supporting Information). Our results clearly demonstrate severe damage only to light‐exposed cells treated with **3M**, which evidently changed their morphology by shrinking and blebbing. The cells that were not treated with **3M** showed no damage under the same laser irradiation. As anthracene analogues upon excitation are known to produce singlet oxygen,^[32]^ we confirmed that **3M** is also able to produce singlet oxygen in solution. The characteristic near‐IR luminescence of singlet oxygen was observed upon excitation of an oxygen‐saturated solution of **3M** in MeCN (Figure 15). The quantum yield for singlet oxygen formation (*Φ*_Δ_) was determined to be 0.2 by comparison to the standard perinaphthenone, which is known to sensitize singlet oxygen with an efficiency close to unity in MeCN.^[58]^ Thus, the photo‐induced cytotoxicity of **3M** is suggested to be due to the photosensitized production of singlet oxygen by compound **3M** inside the cells.

The measurable cytotoxicity suggested efficient cellular uptake of **1M** and **3M**. Thus, to study the chromophore uptake and distribution, and taking advantage of the strong fluorescence of **3M**, we employed confocal microscopy (Figure 14), which showed significant co‐localization of **3M** with LysoTracker that stains acidic organelles, such as lysosomes (Pearson's coefficient=0.68) and negligible co‐localization with MitoTracker that stains mitochondria (Pearson's coefficient=0.19).

## Conclusion

All of the compounds show interesting photophysical properties. The dications show bathochromic shifts in emission compared to the neutral compounds. The neutral compounds show very small Stokes shifts (360–700 cm^−1^), which are larger for the dications (2300–3870 cm^−1^); however, still smaller than those of our related pyrene‐based compounds (4300 cm^−1^).^[16]^ All of the compounds have relatively short fluorescence lifetimes (<4 ns). Compound **3** has a fluorescence quantum yield near unity in CH_2_Cl_2_ and 0.93 in MeCN. The neutral compounds (**1** and **3**) show higher quantum yields than their respective dications. The very low QY (0.03) of **2** is most likely due to vibrational relaxation modes involving the central fluorinated moiety. With stronger acceptors, **3M** showed larger two photon absorption (2PA) cross‐section than **3**. The 2PA properties of **3M** are quite similar to other topologically similar substituted 9,10‐dialkynylanthracene chromophores, for example, triarylamine or dialkylaminophenyl, which represent the popular donor‐acceptor‐donor motif. However, the central anthracene moiety in **3M** acts as the donor in this quadrupolar acceptor‐donor‐acceptor chromophore. All of the dications showed reduction events characteristic of viologen compounds. However, only **3M** showed a reversible cyclic voltammogram.

All of the dications showed interactions with DNA/RNA, demonstrated by several independent methods (UV/Vis, fluorimetric, and CD titrations, thermal denaturation experiments, viscometry study). The smaller arene derivatives (**1M** and **2M)** bind in DNA/RNA grooves, while the larger arene‐analogue **3M** intercalates^[39]^ and, when added in excess over DNA/RNA, **3M** formed aggregates along the polynucleotide. Most intriguingly, **3M** showed a highly specific emission response upon binding to ds‐DNA/RNA, which enforced the planar achiral small molecule into a chiral rotamer, as characterised by its exceptionally strong CD spectrum.

Surprisingly, all of the dications showed only negligible toxicity to human cell lines, although they interact quite strongly with DNA/RNA. Particularly for **3M**, confocal microscopy demonstrated efficient cellular uptake and low bioactivity of such a DNA‐intercalating agent, which was attributed to its accumulation in lysosomes, that prevented its binding to nuclear DNA and consequent toxic effects. However, the observed photo‐induced toxicity of **3M** at 1 μM concentration is attributed to photosensitized production of singlet oxygen.^[39]^ In summary, **3M**, an acceptor‐π‐acceptor system, is not only a promising bio‐active dye as a lead compound for the development of novel photo‐activated theranostic agents, but also shows interesting two‐photon absorption, and redox properties. These intriguing features make **3M** a truly multi‐functional material.

## Experimental Section

**Materials and methods**: The compounds 1,4‐diiodobenzene, 1,4‐diiodotetrafluorobenzene, 9,10‐dibromoanthracene and other reagents were purchased from common commercial sources, and 4‐ethynylpyridine was prepared by literature procedures.^[60]^ Triethylamine was dried using an Innovative Technology Inc. Solvent Purification System, and was further deoxygenated.

^1^H and ^13^C{^1^H} solution NMR spectra were recorded at ambient temperature using a Bruker Avance 300 NMR spectrometer, operating at 300 MHz for ^1^H and 75 MHz for ^13^C{^1^H}. Chemical shifts (δ) were referenced to residual solvent peaks. ^19^F{^1^H} NMR spectra were recorded using a Bruker Avance 500 NMR spectrometer operating at 470 MHz. High resolution mass spectrometry (HRMS) was performed using a Thermo Fisher Scientific Exactive Plus Orbitrap MS System with either an Atmospheric Sample Analysis Probe (ASAP) or an electrospray ionization (ESI) probe. Elemental analyses were performed on an Elementar vario MICRO cube elemental analyser.

Syntheses of **1**–**3** were carried out following slightly modified literature methods^[36]^ as described below.

**Synthesis of 1**: A mixture of 4‐ethynylpyridine (0.60 g, 5.82 mmol), 1,4‐diiodobenzene (0.85 g, 2.58 mmol), [Pd(dppf)Cl_2_] (0.11 g, 0.15 mmol) and CuI (0.03 g, 0.16 mmol) was added to dried and deoxygenated triethylamine (150 mL) under an argon atmosphere. The reaction mixture was stirred for 24 h after which the solvent was removed *in vacuo*. The solid residue was extracted with CH_2_Cl_2_ and the resulting solution was eluted through a short column of alumina (4 cm). Removal of the solvent gave the desired compound as a white powder. Yield 0.60 g (83 %). ^**1**^ **H NMR** (300 MHz, CDCl_3_): *δ* 8.63 (dd, *J*_1_=5 Hz, *J*_2_=2 Hz, 4H), 7.57 (s, 4H), 7.41 (dd, *J*_1_=5 Hz, *J*_2_=2 Hz, 4H). ^**13**^ **C{^1^H} NMR**: *δ* 149.8, 131.9, 131.1, 125.5, 122.9, 93.3, 88.8. **HRMS** (ASAP^+^): *M/Z* found=281.1063; *M/Z* calculated for [M+H]^+^ (C_20_H_13_N_2_^+^)=281.1073.

**Synthesis of 1M**: Compound **1** (0.14 g, 0.50 mmol) was stirred with methyl triflate (160 μL, 0.24 g, 1.46 mmol) in dry toluene (15 mL) over 24 h under an argon atmosphere. The yellow precipitate obtained was isolated by filtration, washed with dry toluene and dried. Yield: 0.25 g (82 %). Thin plate‐like single crystals were obtained by slowly evaporating a MeCN solution. ^**1**^ **H NMR** (300 MHz, CD_3_CN): *δ* 8.58 (d, *J*=7 Hz, 4H), 8.02 (d, *J*=7 Hz, 4H), 7.78 (s, 4H), 4.26 (s, 6H). **HRMS** (ESI^+^): *M/Z* found=155.0727; *M/Z* calculated for [M‐2(OTf)]^2+^ (C_22_H_18_N_2_^2+^)=155.0730. **Elem. Anal**. Calcd. (%) C_24_H_18_F_6_N_2_O_6_S_2_: C 47.37, H 2.98, N 4.60, S 10.54: found: C 46.78, H 3.34, N 4.70, S 10.78.

**Synthesis of 2**: A mixture of 4‐ethynylpyridine (0.60 g, 5.82 mmol), 1,4‐diiodotetrafluorodobenzene (1.00 g, 2.49 mmol), [Pd(dppf)Cl_2_] (0.11 g, 0.15 mmol) and CuI (0.03 g, 0.16 mmol) was added to dried and deoxygenated triethylamine (150 mL) under an argon atmosphere. The reaction mixture was stirred for 36 h after which the solvent was removed *in vacuo*. The solid residue was extracted with CH_2_Cl_2_ and the resulting solution was eluted through a short column of alumina (4 cm). Removal of the solvent gave the desired compound as a brown powder. Yield 0.62 g (70 %). ^**1**^ **H NMR** (300 MHz, CDCl_3_): *δ* 8.69 (dd, *J*_1_=5 Hz, *J*_2_=2 Hz, 4H), 7.46 (dd, *J*_1_=5 Hz, *J*_2_=2 Hz, 4H). ^**19**^ **F{^1^H} NMR**: *δ* −135.6. **HRMS** (ASAP^+^): *M/Z* found=353.0697; *M/Z* calculated for [M+H]^+^ (C_20_H_9_F_4_N_2_^+^)=353.0696. **Elem. Anal**. Calcd. (%) C_20_H_8_F_4_N_2_: C 68.19, H 2.29, N 7.95; found: C 67.72, H 2.37, N 8.10.

**Synthesis of 2M**: Compound **2** (0.18 g, 0.51 mmol) was stirred with methyl triflate (160 μL, 0.24 g, 1.46 mmol) in dry toluene (15 mL) over 24 h under an argon atmosphere. The light brown precipitate obtained was isolated by filtration, washed with dry toluene and dried. Yield: 0.29 g (85 %). Needle like single crystals were obtained by slowly evaporating a MeCN solution. ^**1**^ **H NMR** (300 MHz, CD_3_CN): *δ* 8.68 (d, *J*=7 Hz, 4H), 8.13 (d, *J*=7 Hz, 4H), 4.31 (s, 6H). ^**19**^ **F{^1^H} NMR** (470 MHz, CD_3_CN): *δ*: −79.3 (OTf), −135.6. **HRMS** (ESI^+^): *M/Z* found: 191.0538; *M/Z* calculated for [M‐2(OTf)]^2+^ (C_22_H_14_F_4_N_2_^2+^)=191.0541. **Elem. Anal**. Calcd. (%) C_24_H_14_F_10_N_2_O_6_S_2_: C 42.36, H 2.07, N 4.12, S 9.42: found: C41.63, H 2.29, N 3.73, N 9.15.

**Synthesis of 3**: A mixture of 4‐ethynylpyridine (0.62 g, 6.01 mmol), 9,10‐dibromoanthracene (0.84 g, 2.50 mmol), [Pd(dppf)Cl_2_] (0.11 g, 0.15 mmol) and CuI (0.03 g, 0.16 mmol) was added to dried and deoxygenated triethylamine (150 mL) in a J‐Young's tap flask under an argon atmosphere. The reaction mixture was stirred at 70 °C for 48 h, after which the mixture was cooled to room temperature, and the solvent was removed *in vacuo*. The solid residue was extracted with CH_2_Cl_2_ and the resulting solution was eluted through a short column of alumina (4 cm). Removal of the solvent gave the desired compound as a brown powder. Yield 0.73 g (77 %). ^**1**^ **H NMR** (300 MHz, CDCl_3_): 8.73 (dd, *J*_1_=5 Hz, *J*_2_=2 Hz, 4H‐py), 8.65 (m, 4H−Ar), 7.70 (m, 4H−Ar), 7.63 (dd, *J*_1_=5 Hz, *J*_2_=2 Hz, 4H‐py). ^**13**^ **C{^1^H} NMR** (75 MHz, CDCl_3_): *δ* 150.2, 132.4, 131.4, 127.6, 127.2, 125.6, 118.4, 99.9, 90.8. **HRMS** (ESI^+^): *M/Z* found: 381.1359; *M/Z* calculated for [M+H]^+^ (C_28_H_17_N_2_^+^)=381.1387.

**Synthesis of 3M**: Compound **3** (0.19 g, 0.50 mmol) was stirred with methyl triflate (160 μL, 0.24 g, 1.46 mmol) in dry toluene (15 mL) over 48 h under an argon atmosphere. The dark red precipitate obtained was isolated by filtration, washed with dry toluene and dried. Yield: 0.32 g (90 %). Block‐shaped single crystals were obtained by slowly evaporating a MeCN solution. ^**1**^ **H NMR** (300 MHz, CD_3_CN): *δ* 8.80 (m, 4H−Ar), 8.68 (d, *J*=7 Hz, 4H‐py), 8.32 (d, *J*=7 Hz, 4H‐py), 7.88 (m, 4H−Ar), 4.32 (s, 6H). ^**19**^ **F{^1^H} NMR** (470 MHz, CD_3_CN): *δ*: −79.3 (OTf). **HRMS** (ESI^+^): *M/Z* found: 205.0888; *M/Z* calculated for [M‐2(OTf)]^2+^ (C_30_H_22_N_2_^2+^)=205.0886. **Elem. Anal**. Calcd. (%) C_32_H_22_F_6_N_2_O_6_S_2_: C 54.24, H 3.13, N 3.95, S 9.05: found: C 53.96, H 3.81, N 4.22, S 9.05. Single crystals of **3M’** were obtained by ion‐exchange with [*n*‐Bu_4_N][PF_6_] in MeCN. ^**1**^ **H NMR** (300 MHz, CD_3_CN): *δ* 8.79 (m, 4H−Ar), 8.68 (d, *J*=7 Hz, 4H‐py), 8.33 (d, *J*=7 Hz, 4H‐py), 7.88 (m, 4H−Ar), 4.32 (s, 6H).

**Single crystal X‐ray diffraction**: Single crystals, suitable for X‐ray diffraction analysis, were selected, coated in perfluoropolyether oil under a polarised microscope, and mounted on MiTeGen sample holders. The mounted crystals were cooled by a stream of cold nitrogen using Oxford Cryostreams or Bruker Kryoflex II low‐temperature devices. Diffraction data were collected at 100 K on a Bruker X8‐Apex II 4‐circle diffractometer with a CCD area detector using Mo‐*K*α radiation monochromated by graphite or multi‐layer focusing mirrors. The corrections for the Lorentz‐polarisation and absorption effects were applied on the frames by the Bruker software packages. The structures were solved using the intrinsic phasing method (SHELXT).^[61]^ All non‐hydrogen atoms were refined anisotropically, with hydrogen atoms ‘riding’ in idealised positions, by full‐matrix least squares against *F* ^2^ on all data, using SHELXL^[61]^ software and the Shelxle graphical user interface.^[61]^ Structural information was extracted and graphics were produced using Mercury software.^[62]^ Crystal data and experimental details are listed in Table S1 in the Supporting Information.

**General photophysical measurements**: All measurements were performed in standard quartz cuvettes (1 cm ×1 cm cross‐section) fitted with screw caps. UV‐visible absorption spectra were recorded using an Agilent 8453 diode array UV‐visible spectrophotometer. The emission spectra were recorded using an Edinburgh Instruments FLS920 spectrometer equipped with a cooled red PMT detector from Hamamatsu (R13456‐P), or red‐sensitive photomultiplier (PMT‐R928P), and a near‐IR PMT as detectors, and with double monochromators, operating in right‐angle geometry mode. A 450 W continuous xenon arc lamp (Xe900) was employed as the excitation source. All solutions used in photophysical measurements had concentrations lower than 5×10^−6^ M to eliminate excimer formation during fluorescence measurements.

**Fluorescence quantum yield measurements**: The fluorescence quantum yields were measured using a calibrated integrating sphere (150 mm inner diameter) from Edinburgh Instruments combined with the above FLS920 spectrometer. Unless otherwise stated, the longest‐wavelength absorption maximum was chosen as the excitation wavelength. The corresponding blank solvent was used as a reference for solution‐phase measurements, and BaSO_4_ was used as reference for measurements in the solid state.

**Fluorescence lifetime measurements**: Lifetimes were measured using the Edinburgh Instruments FLS920 and FLS980 spectrometers, using picosecond pulsed diode lasers of 315.8 nm, 472.1 nm and 508.8 nm, as applicable, as the light source in the time‐correlated single‐photon counting (TCSPC) mode. Emission decay was detected with a 3 nm emission‐slit‐band‐width. A 5000 kHz (every 200 ns) pulse was generated. The time range was set to 100 ns, and 1024 channels were used. Instrument response functions (IRFs) were measured from the scatter of an aqueous suspension of Ludox at the same excitation wavelength. A reconvolution‐fit was performed using F900 version 7.2.1 software. The quality of the decay fits was assessed by the calculated values of the reduced *χ* ^2^ and Durbin‐Watson parameters, and visual inspection of the weighted and autocorrelated residuals.

**Two‐photon induced fluorescence spectroscopy**: The 2PA cross‐sections of **3** and **3M** were determined by the two‐photon induced fluorescence technique (2PIF), based on measurements of the sample with respect to a reference compound with a well characterized 2PA cross‐section and spectrum. A Solstice amplified Ti:sapphire laser from Newport Spectra‐Physics operating at 1 kHz repetition rate, delivering 100 fs pulses at 800 nm was used. 70 % of the available energy seeded a tuneable computer‐driven optical parametric amplifier (TOPAS−C, Light Conversion). A parabolic mirror with 15 cm focal length was used to focus the vertically polarized light ca. 10 mm before the sample. This provides an almost constant excitation beam cross section passing through the cuvette in close distance through the sample cuvette wall, where the fluorescence is collected. Upon each acquisition, the excitation energy was varied between 0.2–3 μJ. The 1 cm quartz cuvette was mounted on a cuvette holder in a precision rotation stage, ensuring that excitation and emission collection is carried out under the same conditions for the reference and sample compounds. The emitted fluorescence signal was collected at 90° using an achromatic lens and then directed to a compact CCD spectrometer for detection. The 2PA cross‐section as a function of laser excitation energy can be determined using the following equation:^[63]^

$${\left[\sigma_{\;}^{\left(2PA\right)}\left({\tilde{\nu }}_{ex.}\right)\right]}_{S}={\left[\sigma_{\;}^{\left(2PA\right)}\left({\tilde{\nu }}_{ex.}\right)\right]}_{R}\left(\frac{\Phi_{R}}{\Phi_{S}}\right)\left(\frac{C_{R}}{C_{S}}\right)\frac{{I_{\;}^{\left(2PA\right)}}_{S}}{{I_{\;}^{\left(2PA\right)}}_{R}}$$

where indices S and R denote the sample compound and reference compound, respectively, *C* is the compound's concentration, *I* ^(2PA)^ is the two‐photon induced emission signal, and *Φ* is the fluorescence quantum efficiency which related to the ratio between the fluorescence quantum yields of reference and sample as determined through the 2PIF apparatus. Two‐photon excitation was verified by log‐log plots of fluorescence intensities vs. excitation power at various wavelengths, all giving slopes of 2. Verification of the quadratic dependence between the excitation power and emitted fluorescence is shown in Figure S32. Fluorescein in alkaline water solution was used as the reference.^[64]^ All samples had concentrations in the range 10^−5^–10^−6^ M.

**Electrochemistry**: Cyclic voltammetry measurements were carried out using a Gamry Instruments Reference 600 potentiostat. A standard arrangement of a three‐electrode cell configuration was set up using a platinum disk working electrode, a platinum wire counter‐electrode, and a silver wire, separated by a *Vycor* tip, as the reference electrode. The formal redox potentials were referenced to the ferrocene/ferrocenium ([Cp_2_Fe]^+/0^) redox couple by using ferrocene as the internal standard. [n‐Bu_4_N][PF_6_] was employed as the supporting electrolyte. Compensation for resistive losses (*iR* drop) was considered for all measurements.

**Computational details**: All calculations reported in this work were performed using the *Gaussian09‐D01* suite of programs.^[65]^ The geometries of all the compounds were optimized without symmetry constraints using the PBE0 functional^[66]^ including Grimme's dispersion correction with Becke‐Johnson damping (GD3BJ),^[67]^ in combination with a 6–31G* basis set. Frequency calculations were carried out for all structures to check the nature of the stationary states and the absence of any imaginary frequency to confirm that the optimized geometries were genuine minima on the potential energy hypersurface. The solvent effects were taken into account by means of the polarizable continuum model (PCM).^[68]^ Time‐dependent density functional theory (TD‐DFT) calculations were performed at the same level of theory using the previously optimized geometries. For comparison, computations were also carried out using the CAM‐B3LYP functional^[69]^ molecular orbitals, theoretical UV‐Vis absorption and CD spectra were drawn using the *GaussView* program.^[70]^

## Biological studies

**Cells**: Experiments were performed using two human cell lines, epithelial human lung adenocarcinoma A549 (ATCC® CCL‐185™) and human normal lung fibroblast WI‐38 (ATCC® CCL‐75™). Both cell lines adhere to plastic and glass surfaces and were maintained in the cell culture under the same conditions. Cells were grown in Dulbecco Modified Eagle's Medium (DMEM, Sigma Aldric) supplemented with 10 % of fetal bovine serum (FBS, Sigma Aldrich) and incubated in a cell incubator (Thermo Fischer Scientific) at 37 °C and 5 % CO_2_ in a humified atmosphere.

**Cell viability assays**: Chemical compounds (**1M** and **3M**) were diluted in appropriate volumes of DMSO under sterile conditions, in order to obtain a 10 mM stock solution. Solutions were kept in the dark and stored at 4 °C. For testing the cytotoxic effects of each chemical compound on A549 and WI‐38 cells, diluted solutions were prepared in order to obtain the desired range of concentrations, which were studied using the MTT test.^[57]^ Briefly, cells were seeded in 96‐well tissue culture plates (7×10^3^ cells/well) and, 24 h later, were treated with **1M** and **3M** (concentration range=100–1 μM). Cells treated with the same dilutions of DMSO represented the control, while cells treated only with DMEM (10 % FBS) represented negative control. After 72 h, the medium was removed, 1X MTT solution was added into each well, and the plate was incubated (37 °C, 5 % CO_2_) for 3 h, allowing formazan crystals to form. The resulting MTT‐formazan products were dissolved using DMSO, and their absorbance was measured using a microplate reader at 600 nm. For determining the effect of light exposure, cells were seeded on two plates and treated with working solutions of the compound as previously described.^[71]^ Plates were incubated (37 °C, 5 % CO_2_) for 90 min, allowing compounds to enter the cells. Then, cell culture plates prepared as above were treated with **3M** and irradiated in a Luzchem reactor with visible light range (400–700 nm, 8 lamps, in total 8 W, Dose 50.6 mW m^−2^) ∼18 cm lamp to cell‐plate, at the same time point for three days in a row, with the first exposure to the light on the same day that compounds were added to the cells. Exposure to light was 15 or 30 min per day, while the other cell plate was left in the cell incubator in the dark and served as a control.

**Live cell imaging by confocal microscopy**: Live imaging of the cells treated with **3M** was performed on the A549 cell line. Cells were seeded in Ibidi imaging cell chambers (Ibidi®) in 500 μL of medium, 5×10^4^ cells/well, and left in the cell incubator for 48 h (37 °C, 5 % CO_2_). Subsequently, cells were treated with 10 μM solutions of the respective compound and left in the cell incubator for 90 min to allow the compound to enter the cells. After incubation, the medium was replaced with 500 μL of fresh medium and 500 μL of 50 nM LysoTracker Deep Red solution (Invitrogen, Molecular Probes) or 100 nM MitoTracker (Invitrogen, Molecular Probes) was added to the cells. The cells were incubated for 30 min with LysoTracker or 20 min with MitoTracker (37 °C, 5 % CO_2_), allowing the dye to enter the cells. After incubation, the medium was replaced with 500 μL of fresh medium. Co‐localization of the compounds (*λ*_ex_=457 nm, *λ*_em_=500–600 nm) and lysosomes or mitochondria, respectively (*λ*_ex_=644 nm, *λ*_em_=665 nm) was visualized using Leica SP8 X confocal microscope (Leica Microsystems). Images were processed with the Leica Application Suite X (LAS X, Leica) software platform. Co‐localization was assessed by the Pearson's coefficient. Analysis was done using *ImageJ* software and the appropriate *JACoP* plugin.^[59]^

**Crystal structures**: Deposition Numbers 2080718 (for **1M**), 2080719 (for **2M**), 2080720 (for **3M**), and 2080721 (for **3M’**) contain the supplementary crystallographic data for this paper. These data are provided free of charge by the joint Cambridge Crystallographic Data Centre and Fachinformationszentrum Karlsruhe Access Structures service.

## Conflict of interest

The authors declare no competing financial interest.

1

## Supporting information

As a service to our authors and readers, this journal provides supporting information supplied by the authors. Such materials are peer reviewed and may be re‐organized for online delivery, but are not copy‐edited or typeset. Technical support issues arising from supporting information (other than missing files) should be addressed to the authors.

Supporting Information

Supporting Information

Supporting Information

Supporting Information

Supporting Information

## Data Availability

The data that support the findings of this study are available in the supplementary material of this article.
